# Chemical, Molecular, and Single-nucleus Analysis Reveal Chondroitin Sulfate Proteoglycan Aberrancy in Fibrolamellar Carcinoma

**DOI:** 10.1158/2767-9764.CRC-21-0177

**Published:** 2022-07-18

**Authors:** Adam B. Francisco, Jine Li, Alaa R. Farghli, Matt Kanke, Bo Shui, Paul R. Munn, Jennifer K. Grenier, Paul D. Soloway, Zhangjie Wang, Lola M. Reid, Jian Liu, Praveen Sethupathy

**Affiliations:** 1 Department of Biomedical Sciences, College of Veterinary Medicine, Cornell University, Ithaca, New York.; 2 Division of Chemical Biology and Medicinal Chemistry, Eshelman School of Pharmacy, University of North Carolina, Chapel Hill, North Carolina.; 3 State Key Laboratory of Microbial Resources, Institute of Microbiology, Chinese Academy of Sciences, Beijing, P.R. China.; 4 Genomics Innovation Hub, Biotechnology Resource Center, Cornell University, Ithaca, New York.; 5 Department of Cell Biology and Physiology, School of Medicine, University of North Carolina, Chapel Hill, North Carolina.

## Abstract

Fibrolamellar carcinoma (FLC) is an aggressive liver cancer with no effective therapeutic options. The extracellular environment of FLC tumors is poorly characterized and may contribute to cancer growth and/or metastasis. To bridge this knowledge gap, we assessed pathways relevant to proteoglycans, a major component of the extracellular matrix. We first analyzed gene expression data from FLC and nonmalignant liver tissue (*n* = 27) to identify changes in glycosaminoglycan (GAG) biosynthesis pathways and found that genes associated with production of chondroitin sulfate, but not other GAGs, are significantly increased by 8-fold. We then implemented a novel LC/MS-MS based method to quantify the abundance of different types of GAGs in patient tumors (*n* = 16) and found that chondroitin sulfate is significantly more abundant in FLC tumors by 6-fold. Upon further analysis of GAG-associated proteins, we found that versican (*VCAN*) expression is significantly upregulated at the mRNA and protein levels, the latter of which was validated by IHC. Finally, we performed single-cell assay for transposase-accessible chromatin sequencing on FLC tumors (*n* = 3), which revealed for the first time the different cell types in FLC tumors and also showed that *VCAN* is likely produced not only from FLC tumor epithelial cells but also activated stellate cells. Our results reveal a pathologic aberrancy in chondroitin (but not heparan) sulfate proteoglycans in FLC and highlight a potential role for activated stellate cells.

**Significance:**

This study leverages a multi-disciplinary approach, including state-of-the-art chemical analyses and cutting-edge single-cell genomic technologies, to identify for the first time a marked chondroitin sulfate aberrancy in FLC that could open novel therapeutic avenues in the future.

## Introduction

Fibrolamellar carcinoma (FLC) is a rare form of liver cancer that predominantly afflicts adolescents and young adults (1, 2). Patients with FLC lack standard of care, leaving surgical resection as the primary therapeutic option. Unfortunately, often patients are not eligible for surgery due to metastasis at the time of diagnosis. Paramount to improving patient care is the need to identify molecular pathways that are critical to FLC tumor survival and growth.

FLC is characterized by an approximately 400 kb heterozygous deletion on chromosome 19, which creates the *DNAJB1-PRKACA* (DP) fusion kinase (3). DP is found in more than 80% of patients with FLC (4) and genome-scale analyses have identified widespread alterations in chromatin activity (5) leading to dysregulated genes and noncoding RNAs (6, 7), including miRNAs (8, 9). These studies have highlighted increased activation of progrowth pathways (5) and increased resistance to cell death (10).

A characteristic histologic feature of FLC tumors is the presence of thick, fibrous bands (11). This observation brings into focus the likely importance of the extracellular environment to FLC tumor growth and metastasis, as is the case in many other aggressive cancers as well. Surprisingly though, extracellular matrix composition, including proteoglycans (PG) in the pericellular tumor microenvironment, has not been investigated in FLC.

Altered extracellular matrix composition has been identified in numerous cancer types (12–14). These changes include increased deposition of collagens and fibrillins, matrix remodeling enzymes such as matrix metalloproteinases, and elevated abundance of glycosaminoglycans (GAG) and associated PGs. GAGs and PGs are of notable interest as they contribute to both the structure and mechanics of the tumor stroma and enhance extracellular signaling by sequestering and concentrating soluble growth factors. Through these mechanisms, GAGs and PGs play an important role in the processes of angiogenesis, proliferation, and migration ultimately promoting tumor metastasis (15–18).

GAGs are polysaccharides of varying lengths comprised of repeating disaccharide units (19). The most common GAGs are hyaluronic acid (HA), heparan sulfate (HS), and chondroitin sulfate (CS; ref. 3). HA chains bind to many components of the extracellular matrix, including collagen. Unlike HA, HS and CS, which differ in disaccharide composition and glycosidic bond linkage patterns, are conjugated to specific core proteins, which are then reclassified as PGs (20, 21). PGs share many general characteristics, including domains that bind soluble growth factors and stimulate cellular receptors. In addition, individual PGs can vary in length, and the core proteins can be alternatively spliced, all of which can have affect physiologic processes (22, 23). Finally, polysaccharides can undergo extensive sulfation, the effects of which are essential for these functions (24).

Examples of PGs that have been well studied in the cancer context include HS PG, perlecan (HSPG2), and the CS PG, versican (VCAN). HSPG2 binds multiple FGFs and the VEGF, and is significantly upregulated in many cancers, including melanoma (25, 26), breast carcinomas (27, 28), and glioblastoma (29). Mouse xenograft models of these cancers in which HSPG2 expression has been ablated show reduced tumor volume (16, 28, 30). Through similar mechanisms, VCAN is known to promote the metastasis of prostate and breast cancer by promoting platelet-derived growth factor signaling, interacting with selectins, a family of cellular adhesion molecules, and promoting EGFR signaling (31). The role of PGs in liver cancer, particularly hepatocellular carcinoma (HCC), has been studied extensively and multiple HS PGs have been identified as important contributors to tumor progression (32). However, it is unknown whether these mechanisms are shared with FLC.

In this study, we sought to bridge the important knowledge gap on GAGs/PGs in FLC. Specifically, through the combined use of RNA sequencing (RNA-seq), GAG disaccharide quantification by LC/MS-MS, and single-nucleus assay for transposase-accessible chromatin (snATAC), we quantified HS and CS abundance and interrogated the activity of PGs at single-nucleus resolution in FLC and nonmalignant liver (NML) samples. These studies confirmed that FLC tumor cells preferentially produce CS and that VCAN is one of the primary PGs in FLC. Moreover, we demonstrated that there is more ATAC signal at the VCAN locus in activated hepatic stellate cells than any other cell type.

## Materials and Methods

### Human Samples

Written informed consent was obtained from all individuals and studies were performed in accordance with ethical guidelines established by the U.S. federal policy for the protection of human subjects (U.S. Common Rule). Tumor and adjacent nonmalignant liver samples were collected from patients with FLC by surgeons in accordance with the Institutional Review Board protocols 1802007780, 1811008421 (Cornell University, Ithaca, NY) and 33970/1 [Fibrolamellar Cancer Foundation (FCF)] and provided by the FCF. Patients included male and female subjects and some samples were collected from the same patient. All samples were deidentified before shipment to Cornell.

### PolyA+ RNA Library Preparation and Sequencing

The 27 RNA-seq datasets analyzed in this study were generated previously (5, 33). Frozen tumors underwent physical dissociation using a polytron PT1200 E homogenizer (Thomas Scientific) and total RNA was isolated using the Total RNA Purification Kit (Norgen Biotek) per manufacturer's instructions. RNA purity was quantified with the Nanodrop 2000 (Thermo Fisher Scientific) or Nanodrop One and RNA integrity was quantified with the Agilent 4200 Tapestation (Agilent Technologies). Libraries were prepared by the Cornell Transcriptional Regulation and Expression (TREx) Facility using the NEBNext Ultra II Directional RNA kit (New England Biolabs, E7760). Sequencing was performed at the Genomics Facility in the Biotechnology Research Center at Cornell University (Ithaca, NY) on the NextSeq500 (Illumina).

### Quantitative PCR

Reverse Transcription was performed using the High-Capacity RNA-to-cDNA Kit (Thermo Fisher Scientific). Gene expression was quantified with TaqMan Expression assays on a CFX96 Touch Real-Time System thermocycler (Bio-Rad). Gene expression assays were normalized to the expression of *RPS9*. Individual gene assay IDs: CSGALNACT1 Hs00218054_m1, VCAN Hs0017642_m1, CHST3 Hs01000045_m1, CHST11 Hs00218229_m1, RPS9 Hs02339424_m1. Expression values reported are averaged across at least three (*n* = 3) biological replicates unless otherwise stated in the main text.

### Immunoblot Analysis

FLC and NML tissues were lysed in RIPA buffer containing Halt protease and phosphatase inhibitors (Thermo Fisher Scientific) at 4°C. Lysates were incubated for 30 minutes and centrifuged at 14,000 × *g* for 10 minutes at 4°C. Total protein in the supernatant was quantified using the BCA Protein Assay Kit (Thermo Fisher Scientific). Samples were denatured in NuPAGE LDS Sample Buffer (Thermo Fisher Scientific) containing 5% β-Mercaptoethanol for 10 minutes at 70°C and loaded to a 12% SDS-polyacrylamide gel. After electrophoresis, samples were transferred to polyvinylidene difluoride membrane and blocked in TBS containing 0.5% TWEEN20 (TBST) and 3% BSA for 1 hour at room temperature. Membranes were probed for anti-VCAN GAGβ (1:1,000 dilution, rabbit source, Millipore AB1033) or anti-vinculin (1:10,000 dilution, mouse source VLN01, Thermo Fisher Scientific MA5-11690) overnight at 4°C and then incubated with goat anti-rabbit horseradish peroxidase–linked IgG (1:10,000, Cell Signaling Technology). Membranes were visualized using a ChemiDoc MP (Bio-Rad).

### Immunohistofluorescence Analysis

FLC and NML tissues were formalin fixed at the time of surgery, dehydrated in ethanol, and paraffin embedded for tissue sectioning onto glass slides. Tissue was deparaffinized by two incubations in xylene, followed by one incubation in 1:1 xylene:ethanol (3 minutes per incubation). Tissue was rehydrated by incubation in decreasing concentrations of ethanol: twice in 100%, 95%, 75%, and 50% (3 minutes per incubation). Finally, tissue was incubated in a flushing water bath for 15 minutes. Tissue was then incubated in methanol for 20 seconds, followed by equilibration in PBS for at least 2 minutes. Antigen retrieval was performed by incubating for 20 minutes in preheated 10 mmol/L sodium citrate (pH6.0) buffer containing 0.05% tween-20 (weight/volume) submerged in a boiling water bath. Tissue in citrate buffer was then removed for the bath and allowed to cool for 30 minutes. Tissue was then incubated in PBS containing 0.03% tween-20 for 20 minutes followed by blocking in 10% normal goat serum diluted in PBS containing 0.03% tween-20 for 1 hour. Tissue was then washed in PBS containing 0.03% tween-20 for 2 minutes following overnight incubation at 4°C with anti-VCAN GAGβ (1:100 dilution, rabbit source, Millipore AB1033). Tissue was then washed three times in PBS containing 0.03% tween-20 for 15 minutes followed by secondary antibody staining for 1 hour at room temperature (anti-rabbit Alexafluor 488, 1:1,000 dilution, goat source, Thermo Fisher Scientific A32731). Tissue was washed three times in PBS containing 0.03% tween-20 for 15 minutes followed by a 5-minute incubation with 4′,6-diamidino-2-phenylindole (DAPI) to counterstain cell nuclei. Excess DAPI was washed out with two incubations in PBS for 10 minutes each. Tissue was then dried briefly, a small volume VectaMount (Vector Labs, H-5000) of was added, and a coverslip was mounted. Images were acquired on an Olympus DP80 microscope with the CellSense Dimension software package. All images received equal brightness balancing with ImageJ software.

### Single-nucleus Assay for Transposase-accessible Chromatin

#### Homogenization

We generated six libraries from 70 mg of NML, primary FLC tumor, and metastatic FLC tumor either with or without collagenase treatment. Tissue was first chopped over dry ice and then dissociated with a Dounce homogenizer using a loose pestle in 2 mL of homogenization buffer [1× HB: 320 mmol/L sucrose, 30 mmol/L calcium chloride, 18 mmol/L magnesium acetate, 60 mmol/L tris(hydroxymethy)aminomethane (TRIS) pH 7.8, 0.1 mmol/L ethylenediaminetetraacetic acid (EDTA), 0.1% volume for volume (v/v) nonyl phenopolyethoxylethanol-40 (NP40), 0.1 mmol/L phenylmethylsulfonyl fluoride, 1 mmol/L β-mercaptoethanol, 10 mg/mL collagenase IV] for 15–20 strokes. The homogenate was incubated for 3 minutes on ice and then mixed by pipetting 10 times. A total of 6 mL of ATAC-RSB wash buffer (1× ATAC-RSB: 10 mmol/L TRIS pH 7.4, 10 mmol/L sodium chloride, 3 mmol/L magnesium chloride) was added and the homogenate was mixed by pipetting five times, incubated on ice for 5 minutes, and centrifuged at 500 × *g* for 10 minutes at 4°C, and the supernatant was removed without disrupting pellet. The nuclei were resuspended in 2 mL of ATAC-RSB wash buffer and then dissociated with a Dounce homogenizer using a loose pestle for five strokes, and then a tight pestle for 15–20 strokes, and incubated on ice for 3 minutes. Then 6 mL of ATAC-RSB wash buffer was added mixed by pipetting 10 times and incubated on ice for 3–5 minutes. The suspension was passed through 70-μm cell strainer, centrifuged at 500 × *g* for 10 minutes at 4°C, and the supernatant was removed. The pellet was resuspended in 1 mL OMNI-ATAC buffer [1× OMNI-ATAC: 12.5 mmol/L TRIS pH 7.4, 6.25 mmol/L magnesium chloride, 1.25% v/v dimethylformamide (DMF), 0.125% v/v Tween-20, 0.01% v/v digitonin in 0.4× PBS] and passed through a 30-μm cell strainer. A 10 μL aliquot of nuclei was quantified by double staining with DAPI(10 μL of a 100 μg/mL solution incubated 5–10 minutes at room temperature) and trypan blue (10 μL of 0.4% v/v solution incubated 5 minutes at room temperature). The final nuclei count was adjusted to 300,000 nuclei/mL.

#### Tn5 Storage and Transposome Assembly

Tn5 transposase (4 μmol/L) is stored at −80°C and diluted for usage by adding 0.8 volume of 100% glycerol. Tn5 transposomes were assembled by adding 0.11 volume of barcoded Tn5 adaptors (25 μmol/L stock solution) to Tn5 stock solution. The mixture was incubated at room temperature for 12–24 hours. The transposome (∼2 μmol/L) can be used directly or stored at −20°C.

#### Tagmentation and Sample Processing

Combinatorial single-nucleus barcodes were generated using a strategy modified from ref. 34 and developed in the Genomics Innovation Hub at Cornell. Nuclei suspension (8 μL) was distributed onto 96-well plates and 1 μL of each i5 and i7 transposome (final concentration 400 nmol/L), was added to each well, resulting in 96 combinations of Tn5 barcodesper plate. The tagmentation reaction plate was incubated (30 minutes at 50°C) and the reaction was terminated by adding 10 μL 20 mmol/L EDTA (15 minutes at 37°C). Next, 20 μL of Sorting buffer (1× SB: 1× PBS, 2 mmol/L EDTA, 20 ng/mL BSA) was added to each well and nuclei were repooled into a single sample. Intact nuclei were then stained with DRAQ7 for 15 minutes (ABCam, ab109202), passed through a 30 μm filter, and reisolated by FACS using a FACSMelody instrument (Becton, Dickinson). A 96-well destination PCR plate was preloaded with 10 μL of modified sorting buffer (1× SEB: 10 mmol/L TRIS pH 8.0, 12 ng/μL BSA, 0.05% v/v SDS), 25 nuclei were distributed into each well, and incubated for 10 minutes at 55°C to disrupt Tn5. We added 2.5 μL of 5% v/v Triton-X100 per well to neutralize the SDS prior to PCR. Libraries were amplified 15 cycles with a custom universal P5 primer and a barcoded P7 primer (1 μL of 25 μmol/L primer in 25 μL PCR reaction per well).

#### PCR Cleanup, Size Selection, and Sequencing

All wells were repooled and purified with a MinElute PCR purification kit following the manufacturer's instructions (Qiagen, 28004) and eluted twice with 20 μL of the supplied buffer. The 40 μL elution was further purified and size selected using magnetic solid phase reversible immobilization (35) beads following the manufacturer's instructions (Beckman Coulter, A63880) and eluted in 20 μL. Libraries were sequenced on the HiSeq platform (1 lane) at Novogene.

### ArchR Pipeline for Single-nucleus ATAC (snATAC) Analysis

#### snATAC-seq Preprocessing

Undemultiplexed fastq files were processed using cutadapt (36), UMI tools (37), and custom scripts to parse and assemble combinatorial barcodes from read segments and extract valid single-nucleus barcodes (with error correction). Preprocessed reads were mapped to the human genome (hg38) with bwa mem (38) and duplicates removed with UMI tools. Deduplicated bam files were sorted and indexed using samtools (39) and converted to fragment files using the single-cell analysis tool kit Sinto (https://github.com/timoast/sinto) with –use_chrom “” and –barcode_regex “(?⇐_)(.*)(? = _)” parameters. Fragment files are then sorted and finally used to generate tabix files using tabix (40) prior to loading into ArchR (41).

#### snATAC-seq QC and Dimensionality Reduction and Clustering Analysis

The transcription start site (TSS) enrichment score and fragment number of each nucleus is calculated using ArchR (41) v1.0.1. Nuclei with TSS enrichment score less than 3 and fragment number less than 1,000 are removed. Doublet scores were calculated with default parameters.

We preformed iterative latent semantic indexing by using the “addIterativeLSI” function of ArchR. We then used the default harmony algorithm to correct for batch-effects differences and added clusters using the “addClusters” function.

#### Identification of Marker Features

We identified cluster markers using the function “getMarkerFeatures” with default parameters and then applied the “addImputeWeights” function to impute the weights of markers. We visualize marker features using the ‘plotEmbedding’ function. To plot browser tracks, we used the “plotBrowserTrack” function and arranged track rows from highest to lowest accessibility using the “useGroups” parameters.

#### Identification of Cell Types from snATAC-seq Data

We used unbiased approaches to assign cell-type identity to clusters. A pairwise comparison of ArchR-defined markers and single-cell RNA-seq liver markers revealed high-confidence cell-type assignments for clusters 1–3, and 5–12. Additional Gene Ontology and Kyoto Encyclopedia of Genes and Genomes analyses confirmed several cluster assignments.

#### Plotting Browser Tracks

Accessibility of chromatin surrounding genes of interest is plotted using the default “plotBrowserTrack” function.

### RNA-seq Bioinformatic Analysis

Paired end RNA-seq reads were aligned to the human genome (hg38) using STAR (v2.4.2a) and reads aligning to the transcriptome were quantified using Salmon (v0.6). Differential expression was determined with DESeq2.0 (v1.3) using a model that accounts for sequencing facility as a covariate.

### Statistical Analysis

Statistical comparisons of quantitative PCR and immunoblot results were made using Student *t* test. Significant differences in gene expression were determined using DESeq2.0. Graphs were generated in the R software package and error bars represent the SE.

### Preparation of ^13^C-labeled CS Disaccharide Calibrants

Four ^13^C-labeled CS disaccharides were prepared from three ^13^C-labeled CS 8-mers, including ^13^C-labeled CS-A 8-mer, ^13^C-labeled CS-C 8-mer, and ^13^C-labeled CS-E 8-mer, as described in Supplementary Fig. S1A. The synthesis of 8-mers was completed using the enzymatic approach (42, 43). The only exception from previously published procedures is that a ^13^C-labeled UDP-GlcA was used to replace unlabeled UDP-GlcA during the synthesis to introduce the ^13^C-labeled GlcA residue to the 8-mer products. The synthesis of UDP-[^13^C]GlcA was completed enzymatically from [^13^C]glucose as described previously (44, 45). The structures of 8-mer products were confirmed by electrospray ionization mass spectrometry.

The 8-mers were then subjected to the digestion of recombinant chondroitin ABCase (*Flavobacterium heparinum*) to yield the disaccharides. The chondroitin ABCase digestion solution contained 3.55 mL 8-mers (2 mg/mL), 400 μL enzymatic buffer [100 mmol/L sodium acetate/2 mmol/L calcium acetate buffer (pH 6.0) containing 0.1 g/L BSA], and 50 μL of recombinant chondroitin ABCase (3 mg/mL). The reaction mixture was incubated at 37°C overnight. The extent of reaction completion was monitored by the strong anion exchange chromatography on a Pro Pac PA1 column (9 × 250 mm, Thermo Fisher Scientific) by measuring the absorbance at 232 nm. The purification of ^13^C-labeled CS disaccharides was performed on the Q-Sepharose fast flow column. Mobile phase A was 20 mmol/L NaOAc, pH 5.0 and mobile phase B was 20 mmol/L NaOAc and 1 mol/L NaCl, pH 5.0. The elution gradient with a flow rate of 1 mL/minute was used. The absorbance at 232 nm was scanned and recorded. After purification, the disaccharides were desalted on a Sephadex G-10 column. The quantification of ^13^C-labeled disaccharide calibrants was performed on the basis of the standard curve of commercially available native CS disaccharide standards (Iduron).

### Structure Analysis of ^13^C-Labeled Disaccharide Calibrants

A strong anion exchange column Pro Pac PA1 (9 × 250 mm, Thermo Fisher Scientific) was used to determine the purity of ^13^C-labeled disaccharides after purification. Mobile phase A was 3 mmol/L NaH_2_PO_4_, pH 3.0. Mobile phase B was 3 mmol/L NaH_2_PO_4_ and 2 mol/L NaCl, pH 3.0. The gradient was as follows: 0–20 minutes 0%–20% B, 20–65 minutes 20%–95% B, 65–72 minutes 95% B and 72–75 minutes 95%–100% B with flow rate of 1 mL/minute. The UV absorbance at 232 nm was scanned and recorded. Each disaccharide calibrant was eluted as doublet peaks from ProPac PA1 column. Such profiles are typical for the CS disaccharides due to the chemistry of the anomeric carbon. Both β-anomer and α-anomer are present in the disaccharides. ESI-MS (Thermo Fisher Scientific TSQ Fortis) analysis was used to confirm the molecular weight of each ^13^C-labeled disaccharide. The ESI-MS analysis was performed in the negative-ion mode and with the following parameters: negative-ion spray voltage at 3.0 kV, sheath gas at 15 Arb, ion transfer tube temperature at 320°C and vaporizer temperature at 100°C. The mass range was set at 200–800. The measured molecular weights (MWs) for the ^13^C-labeled CS disaccharide calibrants are: for Di-0S calibrant was 385.2 (Calc MW = 385.1); for Di-4S calibrant was 465.1 (Calc MW = 465.1); for Di-6S calibrant was 465.1 (Calc MW = 465.1); and for Di-4S6S calibrant was 545.2 (Calc MW = 545.0). From our ESI-MS analysis, we did not observe unlabeled CS disaccharides in the calibrants, suggesting that the calibrants had very high isotopic purity that was suited for the quantitative analysis.

### Quantification of the ^13^C-Labeled Disaccharide Calibrants

The CS native disaccharides (Iduron) were dissolved in water and diluted to the concentration of 5, 10, 20, 40, and 80 μg/mL. A total of 50 μL of the diluted CS was injected into HPLC to make the standard curve to quantify the ^13^C-labeled disaccharide calibrants. The stock solutions of four ^13^C-labeled disaccharide calibrants were diluted to 10 or 20 times and then 50 μL was injected to the HPLC analysis. The concentration of the ^13^C-labeled disaccharide calibrants stocks were determined by comparing the peak areas at 232 nm with unlabeled disaccharides.

### Linear Dynamic Range Determination

Individual stock solutions of four CS unlabeled disaccharides (Iduron) were prepared in water at 1 mg/mL. A stock solution of the mixture of the four unlabeled disaccharides, with the final concentration of 0.25 mg/mL for each disaccharide, was obtained by mixing an equal volume of four individual stock solutions. The linear dynamic range of the working solutions was determined by a serial dilution of the mixture stock solution in water to obtain a range of final concentrations (Supplementary Fig. S1B–E). The ^13^C-labeled CS disaccharides were added to the linear dynamic range working solutions as an internal standard mixture stock solution to the final concentration of 40 μg/mL of ^13^C-labeled di-4S, di-6S, and di-4S6S, respectively and 8 μg/mL of di-0S. The linear dynamic range working solutions containing ^13^C-labeled internal standard were freeze dried and reconstituted in the 20-μL mouse plasma. The reconstituted solutions were filtered by passing through a YM-3KDa spin column (Millipore) and washed twice with deionized water to recover the disaccharides in the eluent. The AMAC (2-aminoacridone) derivatization of lyophilized disaccharides was carried out by adding 6 μL of 0.1 mol/L AMAC solution in DMSO/glacial acetic acid (17:3, v/v) and incubating at room temperature for 15 minutes. Then 6 μL of 1 mol/L aqueous sodium cyanoborohydride (freshly prepared) was added to this solution, where AMAC represents AMAC purchased from Sigma-Aldrich. The reaction mixture was incubated at 45°C for 2 hours. After incubation, the reaction solution was centrifuged to obtain the supernatant that was subjected to the LC-/MS-MS analysis.

### LC/MS-MS Analysis

The analysis of AMAC-labeled disaccharides was performed on a Vanquish Flex UHPLC System (Thermo Fisher Scientific) coupled with TSQ Fortis triple-quadrupole mass spectrometry as the detector. The C18 column (Agilent InfinityLab Poroshell 120 EC-C18 2.7 μmol/L, 4.6 × 50 mm) was used to separate the AMAC-labeled disaccharides. Mobile phase A was 50 mmol/L ammonium acetate in water. Mobile phase B is methanol. The elution gradient of from 5%–45% mobile phase B in 10 minutes, followed by isocratic 100% mobile phase B in 4 minutes and then isocratic 5% mobile phase B in 6 minutes was performed at a flow rate of 0.3 mL/minute. Online triple-quadrupole mass spectrometry operating in the multiple reaction monitoring mode was used as the detector. The ESI-MS analysis was operated in the negative-ion mode using the following parameters: negative-ion spray voltage at 4.0 kV, sheath gas at 45 Arb, aux gas 15 arb, ion transfer tube temperature at 320°C and vaporizer temperature at 350°C. TraceFinder software was applied for data processing. The normalized peak areas of the ^13^C-labeled calibrants were plotted against the concentrations of linear dynamic working solutions.

### Analysis of CS and HS from Tissues

CS was extracted from 5 NML and 11 FLC tissues. All tissues were excised, homogenized, and defatted by suspension and vortex in chloroform and methanol mixtures [2:1, 1:1, 1:2 (v/v)]. The defatted tissues were dried and weighed to obtain the dry weight. The dried and defatted tissues were digested with Pronase E [10 mg:1 g (w/w), tissue/Pronase E] at 55°C for 24 hours to degrade the proteins. CS was recovered from the digested solution using a DEAE column. DEAE column mobile phase A was 20 mmol/L Tris, pH 7.5 and 50 mmol/L NaCl, and mobile phase B was 20 mmol/L Tris, pH 7.5 and 1 mol/L NaCl. After loading the digested solution, the column was washed with 10-column volumes of buffer A to discard the contaminants, following by 10 column volumes of buffer B to elute the CS fraction. The CS eluting from the DEAE column was desalted using an YM-3KDa spin column and washed three times with deionized water to remove salt. A known amount of ^13^C-labeled calibrants were added to the digestion solution. The 200 μL of enzymatic buffer [100 mmol/L sodium acetate/2 mmol/L calcium acetate buffer (pH 7.0) containing 0.1 g/L BSA], and the 60 μL of chondroitin ABCase (3 mg/mL) was added to digest the retentate on the filter unit of the YM-3KDa column. The column was incubated at 37°C overnight. The CS disaccharides and calibrants were recovered by centrifugation, and the filter unit was washed twice with 200 μL of deionized water. The collected filtrates were freeze dried before the AMAC derivatization. The AMAC label and LC/MS-MS analysis of the collected disaccharides of tissues was performed as described above. The amount of tissue CS was determined by comparing the peak area of native disaccharide to each calibrant. HS was extracted from two NML tissues and four FLC tissues. The method for the analysis of HS followed the procedures described in a previous publication (46). Three CS disaccharides, including Ddi-2S, Ddi-2S6S, and Ddi-2S4S, were only subjected to relative quantitation as the ^13^C-labeled disaccharides were unavailable. Standard curves of these three disaccharides were generated using unlabeled disaccharide standards that were purchased from Iduron.

### Data Availability

Previously published (33) RNA-seq data can be downloaded from Gene Expression Omnibus (GEO) using the following GEO accession number: GSE181922. Previously published (5) RNA-seq and chromatin run-on sequencing (ChRO-seq) can be downloaded from the European Genome-Phenome Archive (EGA) using the following EGA accession number: EGAS00001004169. Single-nucleus assay for transposase-accessible chromatin followed by sequencing (snATAC-seq) bam and tabix data files generated in this study have been deposited in the GEO and are accessible through the accession number GSE202315.

### Schematics

Chemical structures were created with Chemdraw (by PerkinElmer). Schematics were created with BioRender.com.

## Results

### Chondroitin but not HS Biosynthesis Genes are Increased in FLC

Tissue samples from patients with FLC were acquired at the time of surgical procedures through a collaboration with the FCF and subjected to RNA extraction and messenger RNA-seq (*n* = 23 tumor samples and *n* = 4 adjacent NML samples), as reported previously (5). We then performed an analysis of differential gene expression for enzymes related to GAG biosynthesis, focusing on HA, HS, and CS (3).

**FIGURE 1:**
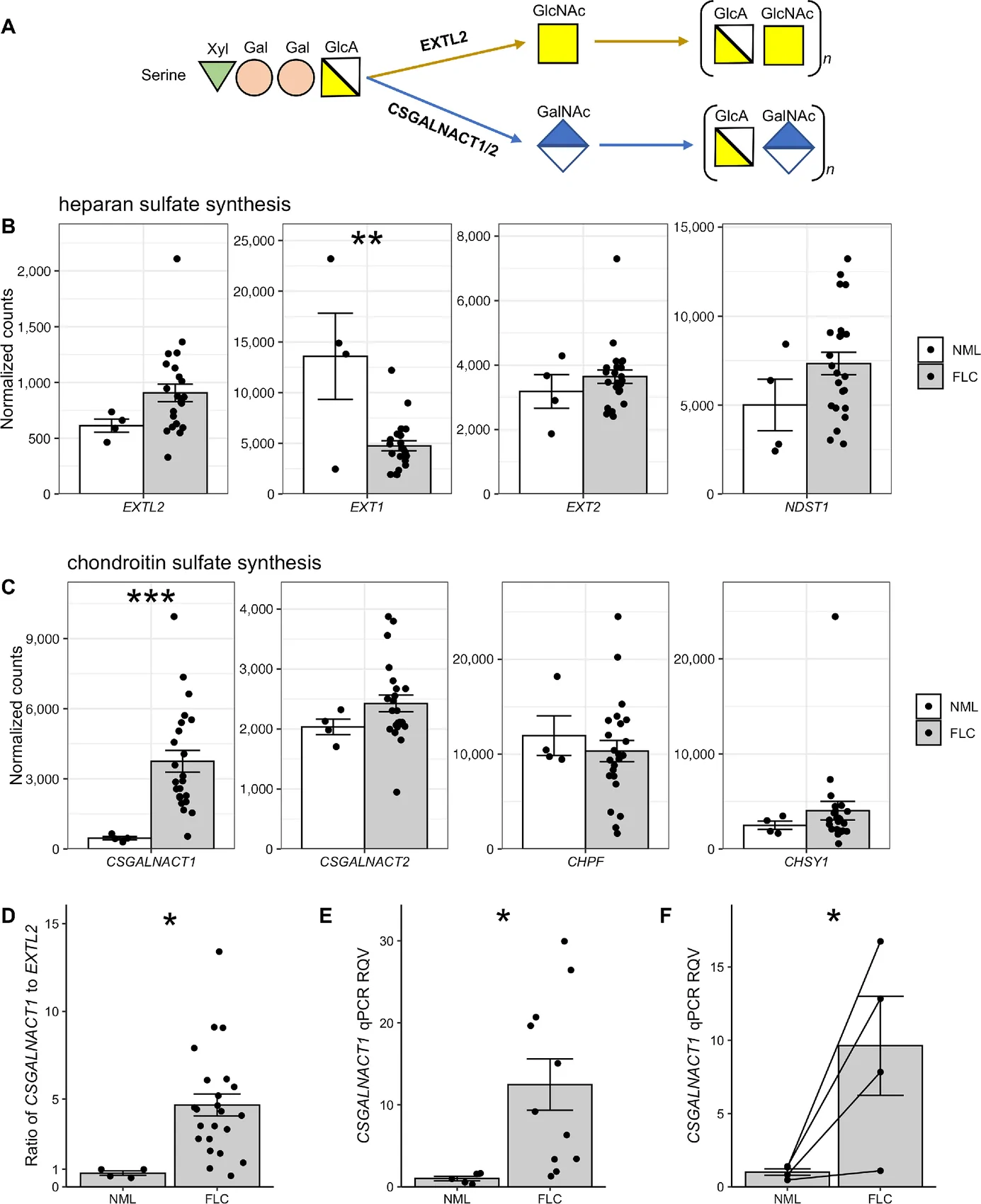
*CSGALNACT1* is dramatically upregulated in FLC. **A,** A schematic showing the sequence of events leading to heparan and chondroitin synthesis. **B,** Differential expression of *EXTL2*, *EXT1*, *EXT2*, and *NDST1* is shown as normalized counts in FLC (*n* = 23) and NML (*n* = 4). **C,** Differential expression of *CSGALNACT1*, *CSGALNACT2*, *CHPF*, and *CHSY1* is shown as normalized counts in FLC (*n* = 23) and NML (*n* = 4). **D,***CSGALNACT1* expression relative to *EXTL2* expression is shown as a ratio of normalized counts in FLC (*n* = 23) and NML (*n* = 4). **E,** Quantitative PCR showing the relative quantitative value (RQV) of *CSGALNACT1* in a separate cohort of FLC samples (*n* = 11) compared with NML samples (*n* = 5). **F,** Quantitative PCR showing the RQV of *CSGALNACT1* in a subset of FLC samples (*n* = 4) that have matched NML tissue. The matched NML/FLC samples are indicated with a line linking the two data points. *, *P* < 0.05; **, *P* < 0.01; ***, *P* < 0.001, two-tailed Student *t* test.

The expression of HA synthase 1–3 in FLC did not meet our standard threshold of robust expression (>500 normalized counts), and therefore the HA pathway was not considered for further analysis. We then assessed the expression of genes which catalyze the formation of the common tetrasaccharide linker required for HS and CS PG production (Fig. 1A). The initial addition of xylose to serine residues in polypeptide chains is catalyzed by xylosyltransferases (*XYLT1* or *XYLT2*; ref. 47). This reaction is followed first by the addition of two galactose molecules [catalyzed by β1,4-galactosyltransferase-I (*B4GALT7*) and β1,3-galactosyltransferase-I (*B3GALT6*)], and subsequently by the addition of glucuronic acid [catalyzed by β1,3-glucuronyltransferase- I (*B3GALT3*)] (48, 49). We found that the expression in FLC of the genes that code for these enzymes meets the threshold criteria but is not significantly different relative to NML.

We next interrogated those genes encoding enzymes responsible for HS and CS polymerization. HS chain formation begins with the addition of *N*-acetylglucosamine to the common linker by *N*-acetylglucosaminyltransferase (*EXTL2*), followed by the addition of glucuronic acid (GlcA) by exostosin glycosyltransferases (*EXT1* and *EXT2*) to create the HS disaccharide (ref. 20; Fig. 1A). We found that the expression of *EXTL2* and *EXT2* is unchanged in FLC, and that *EXT1* is significantly decreased (Fig. 1B). In addition, HS chains undergo deacetylation and sulfation, catalyzed by the enzyme N-deacetylase and N-sulfotransferase 1 (*NDST1*; ref. 50), as a critical maturation step and there is no significant change in the expression of this gene in FLC (Fig. 1B).

CS chain elongation begins with the addition of *N*-acetylgalactosamine (GalNAc) to the common linker by CS GalNAc transferase 1 or 2 (*CSGALNACT1* and *CSGALNACT2*), followed by the addition of GlcA by an enzyme complex containing chondroitin synthase 1 (*CHSY1*) and chondroitin polymerizing factor (CHPF) to create the CS disaccharide (ref. 51; Fig. 1A). We found that the expression of *CSGALNACT1* is significantly increased (8-fold, adjusted *P* = 7.6 × 10^−9^) in FLC compared with NML (Fig. 1C). The expression levels of *CSGALNACT2*, *CHPF*, and *CHSY1* are unchanged in FLC; however, *CHPF* is more abundant in FLC than any of the HS polymerizing factors (Fig. 1C).

Given that HS and CS chains share a common linker, the stoichiometric ratio of *EXTL2* and *CSGALNACT* enzymes is the primary factor determining whether HS or CS chains will be generated (52). We compared the expression of *CSGALNACT1* with *EXTL2* within each of the FLC and NML samples and found that *CSGALNACT1* is on average approximately 4.5 times (*P* = 0.017) more abundant than *EXTL2* (Fig. 1D) in FLC samples, while being roughly equal in NML samples (0.77-fold). In an independent cohort of patients (FLC *n* = 11 and NML *n* = 4), we confirmed by qRT-PCR that *CSGALNACT1* expression is increased >10-fold (Fig. 1E). A similar result was obtained when the analysis was restricted only to matched patient samples (Fig. 1F).

### CS Chains are Aberrantly Elevated in FLC

**FIGURE 2:**
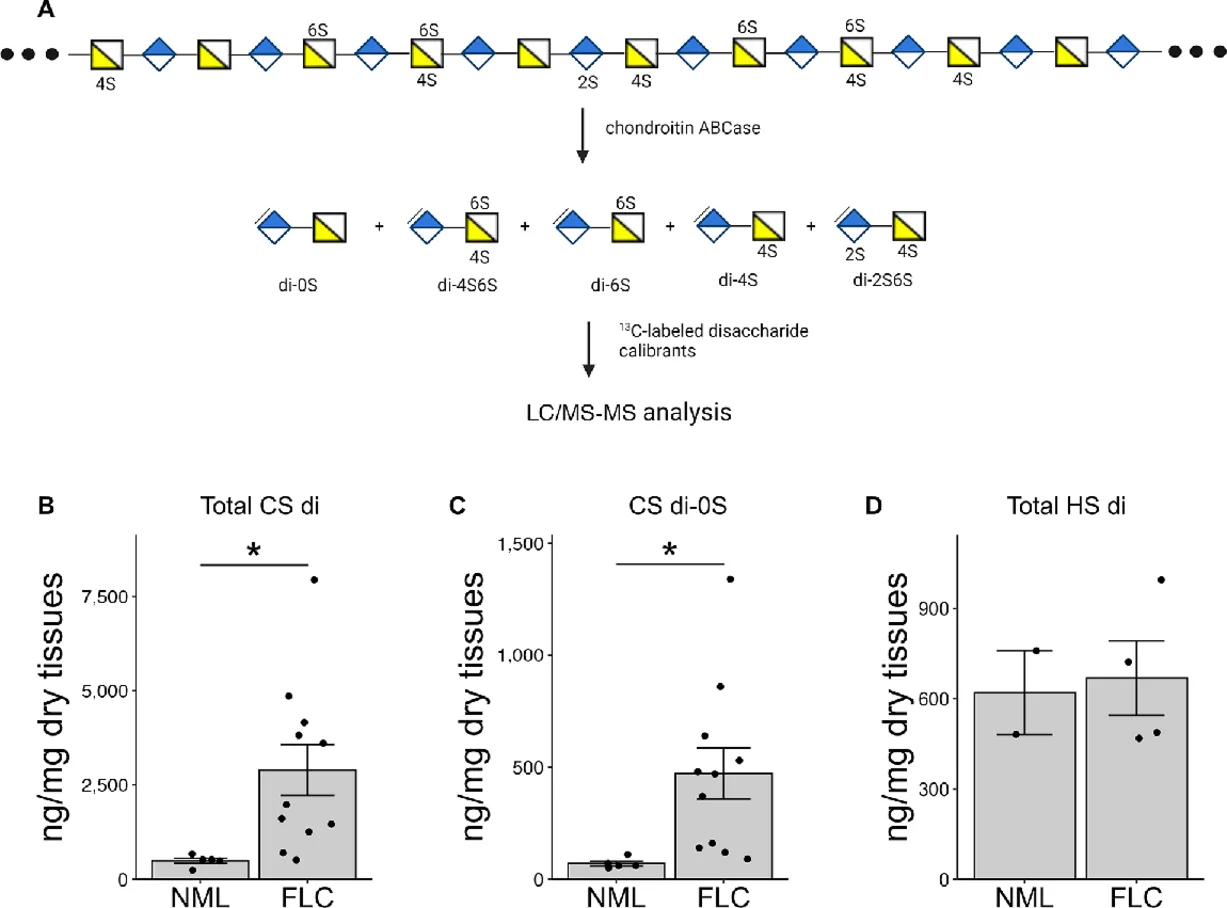
CS disaccharide abundance is significantly increased in FLC. **A,** A schematic diagram detailing disaccharide extraction and identification. **B,** Nanograms of CS (3) per milligram of dry tissue in FLC (*n* = 11) and NML (*n* = 5) tissue. **C,** Nanograms of non-sulfated CS (CS di-0S) per milligram of dry tissue in FLC (*n* = 11) and NML (*n* = 5) tissue. **D,** Nanograms of HS per milligram of dry tissue in FLC (*n* = 4) and NML (*n* = 2) tissue. *, *P* < 0.05, two-tailed Student *t* test.

The gene expression analysis is strongly suggestive of increased CS, but not HS, abundance in FLC. To test this hypothesis, we quantified HS and CS abundance in FLC using a novel chemical analytic method. Because of the relatively low abundance of CS from biological tissues, a new quantitative CS analytic method with high sensitivity was developed for this study. Disaccharide analysis is a commonly used approach to analyze the structure of CS polysaccharides. The method involves the degradation of CS polysaccharides into disaccharides using chondroitin ABCase, and the resultant disaccharides were subjected to LC/MS-MS analysis (Fig. 2A). Furthermore, summing up the amounts of individual disaccharides from the digested CS provides the total amount of CS. To increase the quantitation capability, we employed four ^13^C-labeled CS disaccharide calibrants as internal standards, including di-0S, di-4S, di-6S, and di-4S6S (Supplementary Fig. S1). The ^13^C-labeled CS disaccharide calibrants were obtained from three uniquely designed ^13^C-labeled CS octasaccharides (8-mers) that were synthesized by an enzymatic approach. The di-4S disaccharide is found in CS-A polysaccharide, whereas di-6S and di-4S6S are found in CS-C and CS-E polysaccharides, respectively. The di-0S disaccharide is found in all subtype CS polysaccharides from biological sources. The inclusion of ^13^C-labeled calibrants eliminated batch-to-batch variations, increasing the data consistency (Supplementary Fig. S1B–E).

Using this highly sensitive method, we discovered that total CS in FLC tumor tissue compared with NML (FLC = 11, NML = 5) was significantly increased (5.9-fold, *P* = 0.033; Fig. 2B) and the amount of non-sulfated CS (di-0S) is also similarly increased (6.7-fold, *P* = 0.035) in FLC (Fig. 2C). However, there was no difference in total HS in a subset of FLC tumors (FLC = 4, NML = 2; Fig. 2D).

**FIGURE 3:**
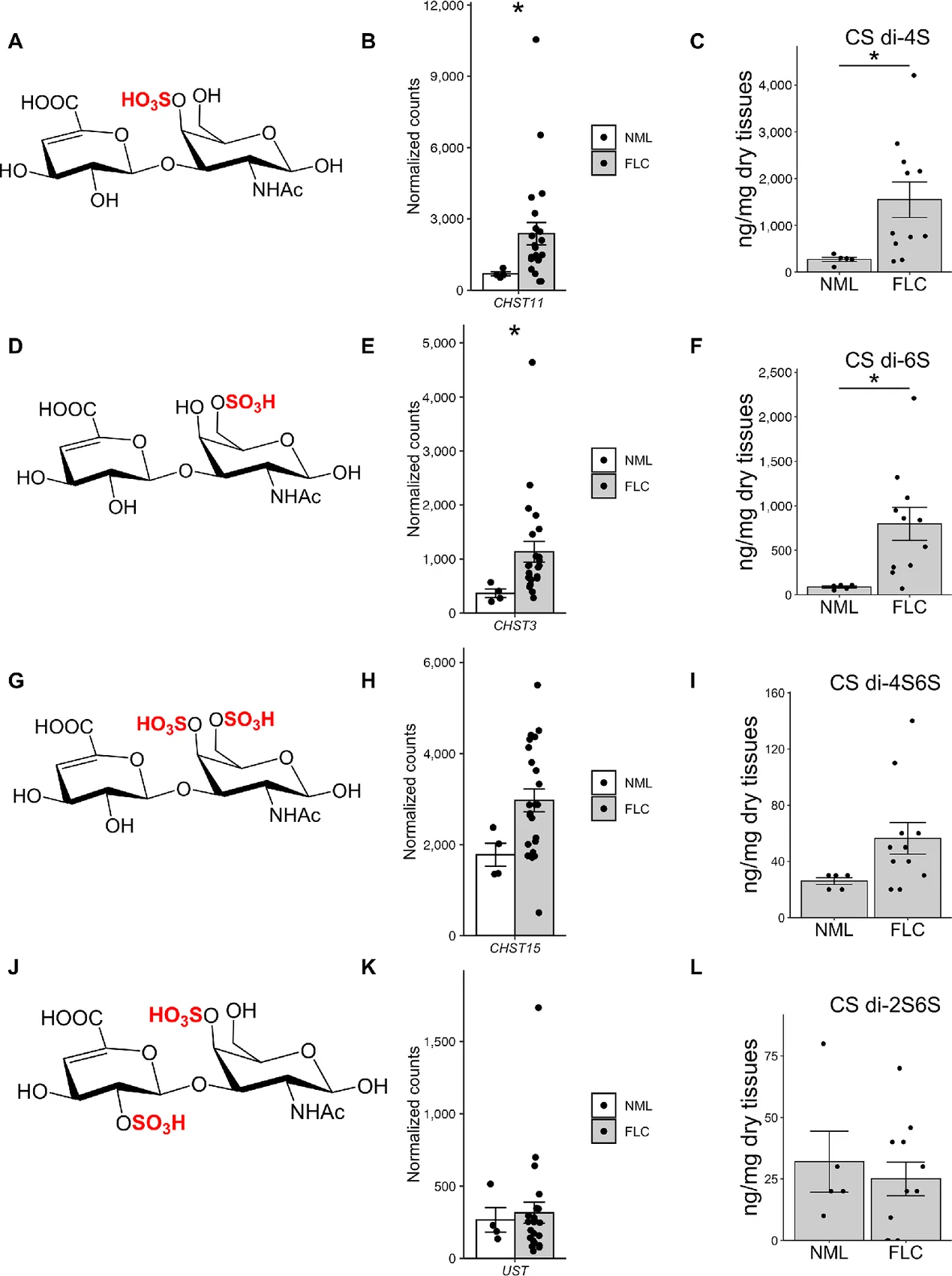
Monosulfated CS disaccharide abundance is significantly increased in FLC. **A,** A schematic diagram detailing CS di-4S. **B,** Differential expression of *CHST11* is shown as normalized counts in FLC (*n* = 23) and NML (*n* = 4). **C,** Nanograms of CS di-4S per milligram of dry tissue in FLC (*n* = 11) and NML (*n* = 5) tissue. **D,** A schematic diagram detailing CS di-6S. **E,** Differential expression of *CHST3* is shown as normalized counts in FLC (*n* = 23) and NML (*n* = 4). **F,** Nanograms of CS di-6S per milligram of dry tissue in FLC (*n* = 11) and NML (*n* = 5) tissue. **G,** A schematic diagram detailing CS di-4S6S. **H,** Differential expression of *CHST15* is shown as normalized counts in FLC (*n* = 23) and NML (*n* = 4). **I,** Nanograms of CS di-4S6S per milligram of dry tissue in FLC (*n* = 11) and NML (*n* = 5) tissue. **J,** A schematic diagram detailing CS di-2S4S. **K,** Differential expression of *UST* is shown as normalized counts in FLC (*n* = 23) and NML (*n* = 4). **L,** Nanograms of CS di-2S4S per milligram of dry tissue in FLC (*n* = 11) and NML (*n* = 5) tissue. *, *P* < 0.05, two-tailed Student *t* test.

The sulfation of CS chains is mediated by a family of chondroitin sulfotransferase (30) enzymes that have specificity for particular positions of oxygen on the CS disaccharide. We interrogated the expression of key *CHST* genes in FLC and measured the abundance of sulfated CS. Monosulfation of the 4-OH position of the GalNAc residue (CS di-4S; Fig. 3A) is catalyzed by chondroitin 4-*O* sulfotransferase (*CHST11*), and we found that the expression of this gene is significantly increased in FLC (2.6-fold, *P*_adjusted_ = 0.03; Fig. 3B). Correspondingly, we also found that CS di-4S is highly elevated in FLC (5.7-fold, *P* = 0.043; Fig. 3C). A second common site for monosulfation is at the 6-OH position of the GalNAc residue (CS di-6S; Fig. 3D), which is catalyzed by chondroitin 6-*O* sulfotransferase (*CHST3*). We found that although the expression of *CHST3* in FLC is lower than that of *CHST11*, its levels are also significantly increased compared with NML (2.3-fold, *P*_adjusted_ = 0.02; Fig. 3E). Likewise, although CS di-6S is not as abundant as CS di-4S, it is similarly increased in FLC (9-fold, *P* = 0.023; Fig. 3F).

CS chains with the disaccharide repeat of di-4S can serve as a substrate for further sulfation. CS di-4S6S (Fig. 3G) is generated by GalNAc 4-sulfate 6-*O*-sulfotransferase (*CHST15*) activity. We found that the expression of *CHST15* is modestly, but not significantly, increased (Fig. 3H) and that the abundance of CS di-4S6S is modestly, but not significantly, increased (Fig. 3I). CS di-2S4S (Fig. 3J) is generated by uronyl 2-*O*-sulfotransferase (*UST*) activity. We found that the expression of *UST* is unchanged (Fig. 3K) and the abundance of CS di-2S4S is unchanged (Fig. 3L) in FLC. We performed a similar quantification for HS and found that none of the sulfated subtypes are significantly altered in FLC (Supplementary Fig. S2A–G), consistent with the finding that total HS abundance is unchanged in FLC (Fig. 1D). In an independent patient cohort, we found by qRT-PCR analysis that the expression of *CHST11* is significantly increased in FLC in all samples (Supplementary Fig. S3A) as well as in matched samples only (Supplementary Fig. S3B) and that *CHST3* is trending upward in FLC (Supplementary Fig. S3C and D). Taken together, these molecular and chemical findings strongly indicate that FLC tumors are marked by aberrant levels of total CS as well as specific sulfated subtypes.

### VCAN is the Primary CS-associated Protein in FLC

**FIGURE 4:**
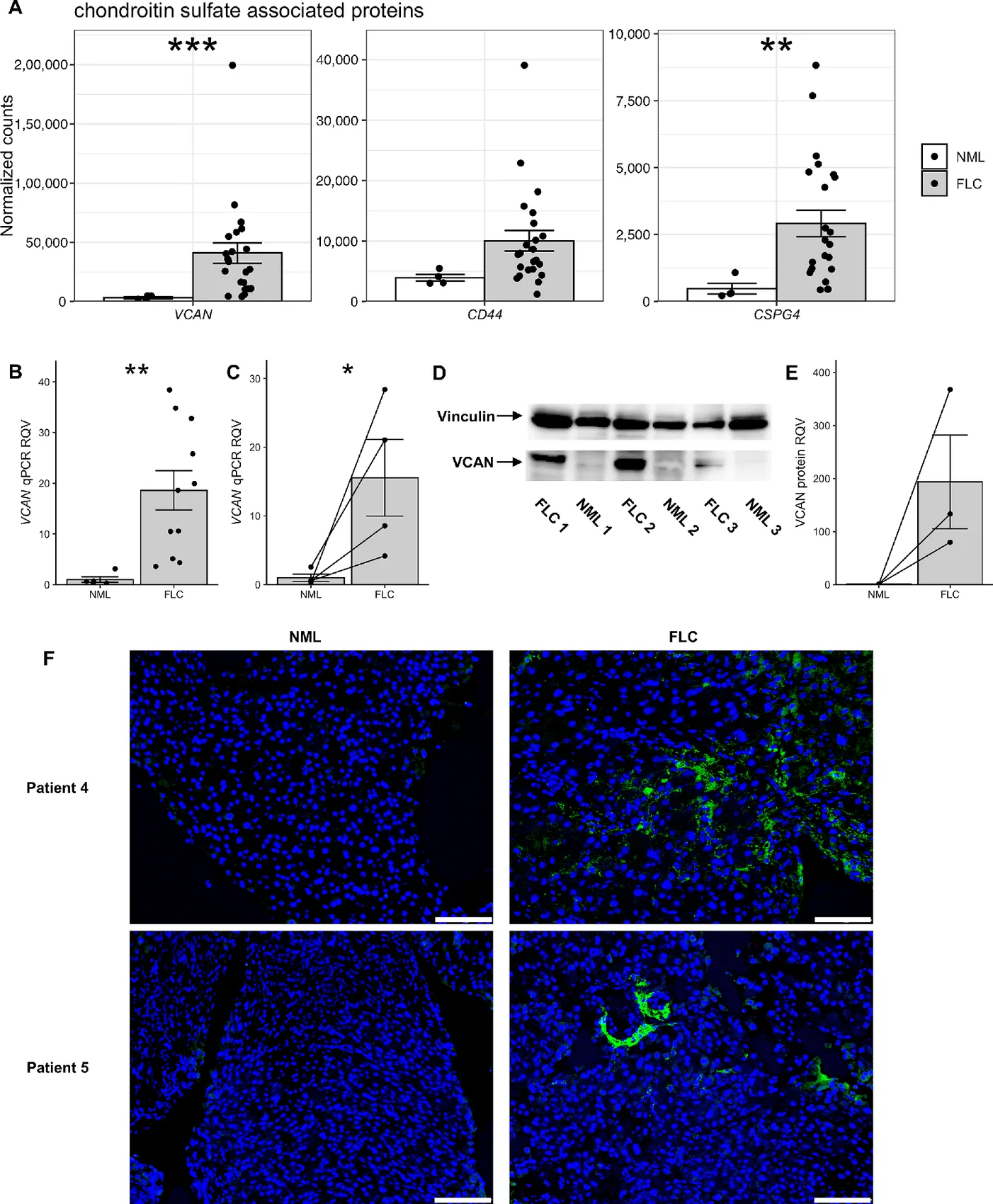
CSAP VCAN is aberrant in FLC. **A,** Differential expression of *VCAN*, *CD44*, and *CSPG4* is shown as normalized counts in FLC (*n* = 23) and NML (*n* = 4). **B,** Quantitative PCR showing the RQV of VCAN in a separate cohort of FLC samples (*n* = 11) compared with NML samples (*n* = 5). **C,** Quantitative PCR showing the RQV of *VCAN* in a subset of FLC samples that have matched NML tissue (*n* = 4). The matched NML/FLC samples are indicated with a line linking the two data points. **D,** Immunoblot in matched FLC/NML samples (*n* = 3) showing VCAN in the lower and vinculin in the top. **E,** VCAN protein levels normalized to vinculin levels are shown as RQV. The matched NML/FLC samples are indicated with a line linking the two data points. **F,** IHF of VCAN protein in two independent patient-matched tissue samples is shown in green. Nuclei are blue. Scale bar, 100 μm. *, *P* < 0.05; **, *P* < 0.01; ***, *P* < 0.001, two-tailed Student *t* test.

We next assessed changes in the expression of CS-associated proteins (CSAP) in FLC. We identified three CSAPs significantly upregulated in FLC compared with NML: chondroitin sulfate proteoglycan 8 (*CSPG8*, also known as *CD44*), chondroitin sulfate proteoglycan 4 (*CSPG4*), and *VCAN*. Notably, the fold change and abundance of *VCAN* in FLC is substantially greater than the other two (*VCAN* ∼10-fold, *P* = 8.5 × 10^−6^; *CD44* ∼2-fold, *P* = 0.11; *CSPG4* ∼ 4.5-fold, *P* = 0.0016; Fig. 4A). We measured *VCAN* by qRT-PCR in an independent FLC patient cohort and observed a significant increase in FLC in all samples (18.5-fold, *P* = 0.0095; Fig. 4B) as well as in matched samples only (15.5-fold, *P* = 0.04; Fig. 4C). Western blot analysis of three matched FLC/NML pairs confirmed dramatic elevation of VCAN protein in FLC (average ∼200-fold; Fig. 4D and E). Specifically, VCAN protein is variable but abundant in the tumor tissue from all 3 patients with FLC, while virtually absent in the adjacent nonmalignant samples (Fig. 4D, bottom). Finally, we performed immunohistofluorescent (IHF) staining on two matched FLC/NML pairs of samples and confirmed that VCAN protein is robustly, though nonuniformly, detected only in tumor tissue (Fig. 4F).

### CS GalNAc Transferase 1 and VCAN are More Altered in FLC Than in Most Other Cancer Types and Correlate with DNAJB1-PRKACA Levels

**FIGURE 5:**
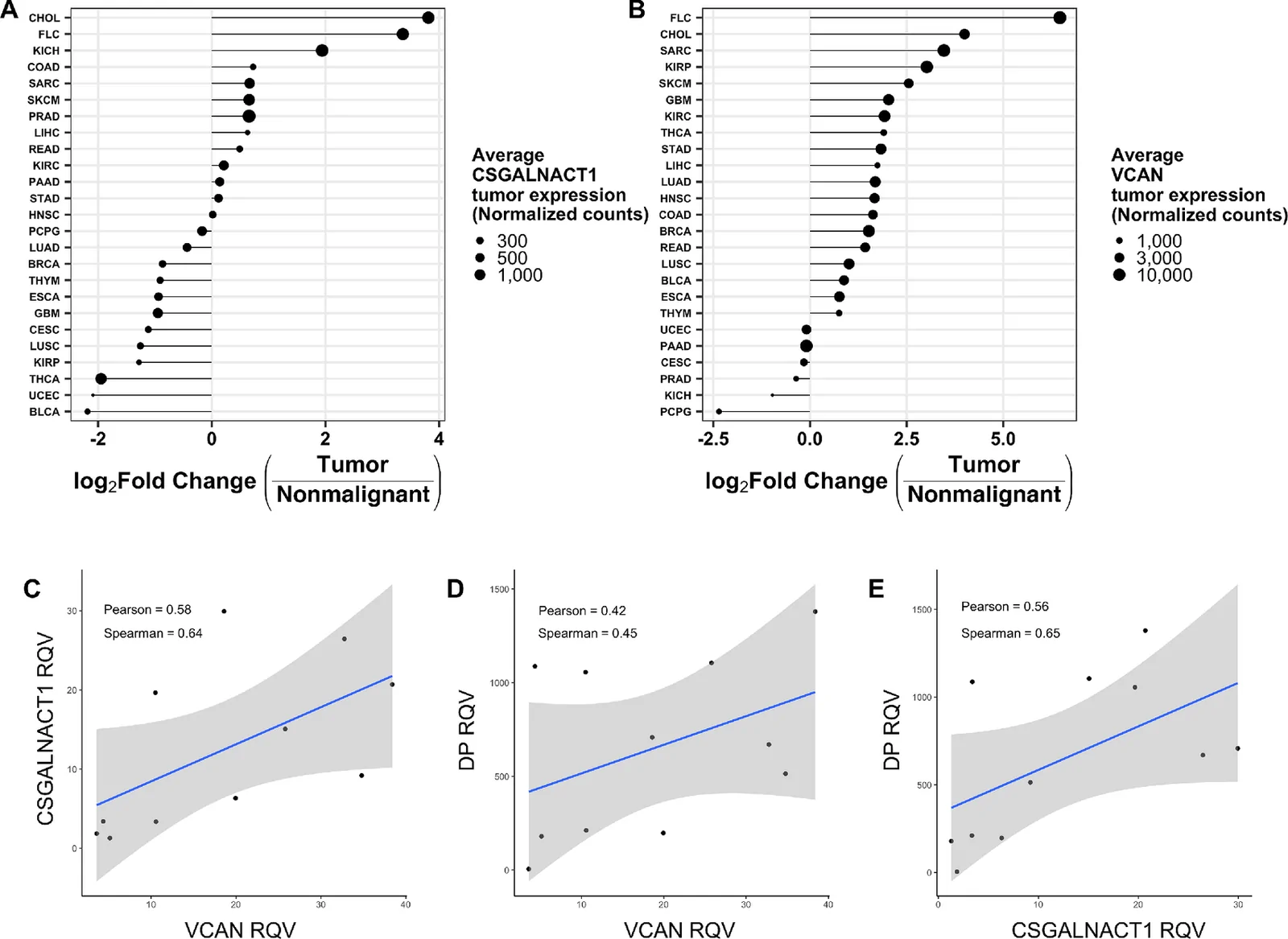
*CSGALNACT1* and *VCAN* are more highly upregulated in FLC than in almost all other cancers and correlate with *DNAJB1-PRKACA* levels. **A,** Normalized counts of *CSGALNACT1* expression in RNA-seq datasets available for 25 tumor types in TCGA. **B,** Normalized counts of *VCAN* expression in RNA-seq datasets available for 25 tumor types in TCGA. **C,** Correlation between *CSGALNACT1* (*y*-axis) and *VCAN* (*x*-axis) shown as the relative quantitative values from qPCR in a separate cohort of FLC samples (*n* = 11). **D,** Correlation between *DNAJB1-PRKACA* (*y*-axis) and *VCAN* (*x*-axis) shown as the relative quantitative values from qRT-PCR in FLC samples (*n* = 11). **E,** Correlation between *DNAJB1-PRKACA* (*y*-axis) to *CSGALNACT1* (*x*-axis) shown as the relative quantitative values from qRT-PCR in FLC samples (*n* = 11). ACC, adenoid cystic carcinoma; BLCA, bladder urothelial carcinoma; BRCA, breast invasive carcinoma; CESC, cervical squamous cell and endocervical adenocarcinoma; CHOL, cholangiocarcinoma; COAD, colon adenocarcinoma; DLBC, diffuse large B-cell lymphoma; ESCA, esophageal carcinoma; FCL, fibrolamellar carcinoma samples analyzed in this study; GBM, glioblastoma; HNSC, head and neck squamous cell carcinoma; KICH, kidney chromophobe; KIRC, kidney renal papillary cell carcinoma; KIRP, kidney renal clear cell carcinoma; LAML, acute myeloid leukemia; LGG, lower grade glioma; LIHC, liver hepatocellular carcinoma; LUAD, lung adenocarcinoma; LUSC, lung squamous cell carcinoma; MESO, mesothelioma; PAAD, pancreatic adenocarcinoma; PCPG, pheochromocytoma and paraganglioma; PRAD, prostate adenocarcinoma; READ, rectum adenocarcinoma; SARC, sarcoma; SKCM, skin cutaneous melanoma; STAD, stomach adenocarcinoma; TGCT, testicular germ cell tumor; THCA, thyroid carcinoma; THYM, thymoma; UCEC, uterine corpus endometrial carcinoma; UCS, uterine carcinosarcoma; UVM, uveal melanoma.

Next, we sought to compare the expression of *CSGALNACT1* and *VCAN* in FLC with other cancer types. Specifically, we queried The Cancer Genome Atlas (TCGA) database, which houses RNA-seq data from 25 other cancer types. We found that the change in expression of *CSGALNACT1* in FLC (relative to corresponding nonmalignant tissue) is second only to cholangiocarcinoma (CCA; Fig. 5A). Strikingly, the change in expression of *VCAN* is greatest in FLC, followed by CCA (Fig. 5B). Further analysis in an independent cohort revealed that *VCAN* and *CSGALNACT1* are correlated (Fig. 5C). In addition, we found by qRT-PCR that the expression of DP correlates with both *VCAN* (Fig. 5D) and *CSGALNACT1* (Fig. 5E).

### VCAN is Expressed in FLC Transformed Epithelial and Tumor-associated, Activated Stellate Cells in FLC

**FIGURE 6:**
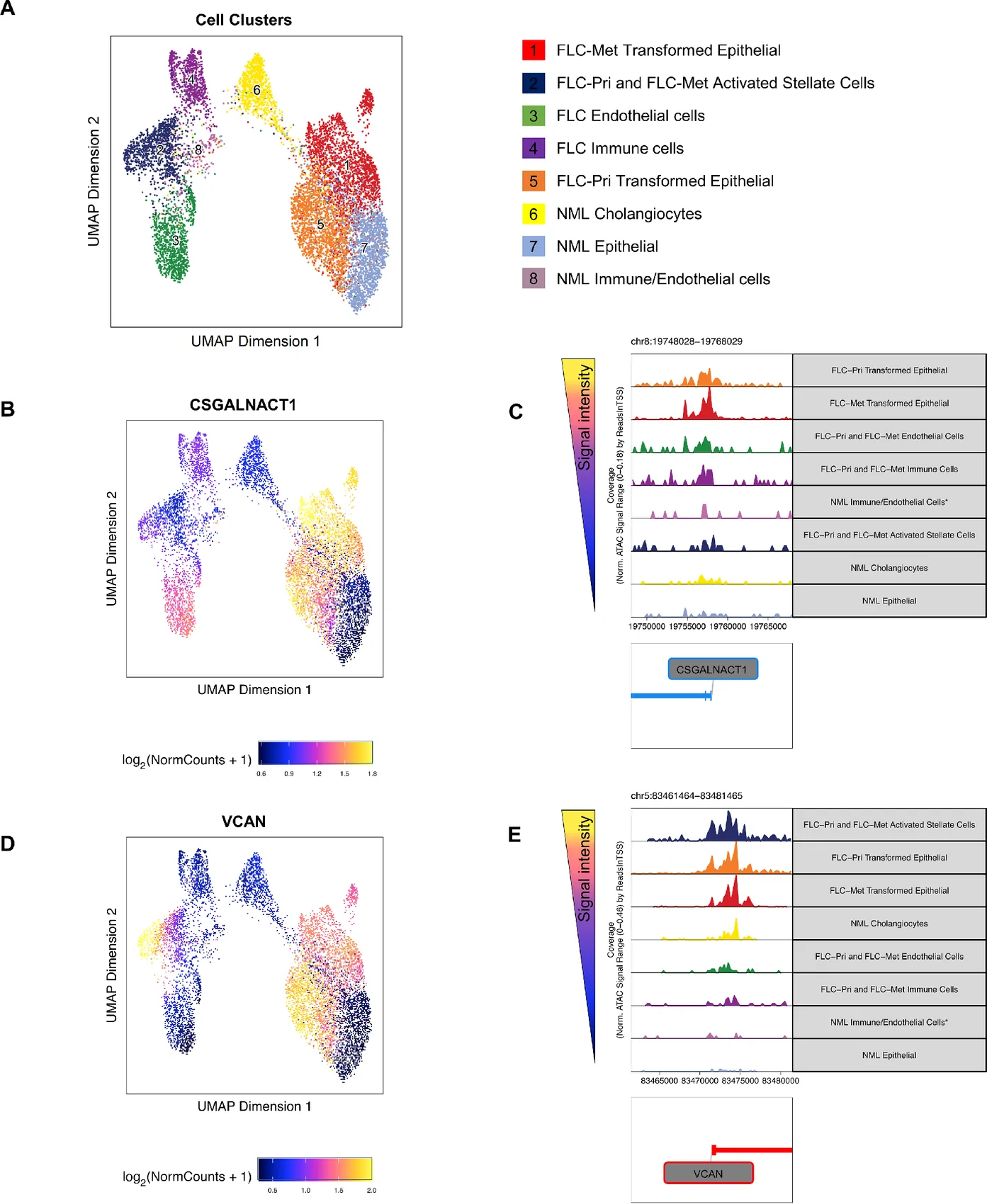
Activity at the *VCAN* locus is high in both tumor epithelial and activated stellate cells in FLC. **A,** UMAP dimensional reduction showing eight cell clusters found in primary FLC, metastatic FLC, and NML tissue (∼9,500 nuclei). **B,** Single-nucleus analysis of chromatin accessibility near the *CSGALNACT1* locus. Increasing signal is indicated by the color gradient (maximum signal is yellow and minimal signal is dark blue). **C,** Genome tracks showing the location of open chromatin near the *CSGALNACT1* locus in each cell type. The annotated transcriptional start site for *CSGALNACT1* is shown at the bottom of the panel. **D,** Single-nucleus analysis of chromatin accessibility near the *VCAN* locus. Increasing signal is indicated by the color gradient (maximum signal is yellow and the minimal signal is dark blue. **E,** Genome tracks showing the location of open chromatin signal near the *VCAN* locus in each cell type. The annotated transcriptional start site for *VCAN* is shown at the bottom of the panel. Cell clusters are denoted through color coding: NML hepatocytes in light blue, FLC primary tumor transformed epithelial cells in orange, FLC metastatic tumor transformed epithelial cells in red, NML cholangiocytes in yellow, FLC primary and metastatic activated stellate cells in dark blue, FLC primary and metastatic immune cells in dark purple, FLC primary and metastatic endothelial cells in green, and NML immune and endothelial cells in light purple.

**FIGURE 7:**
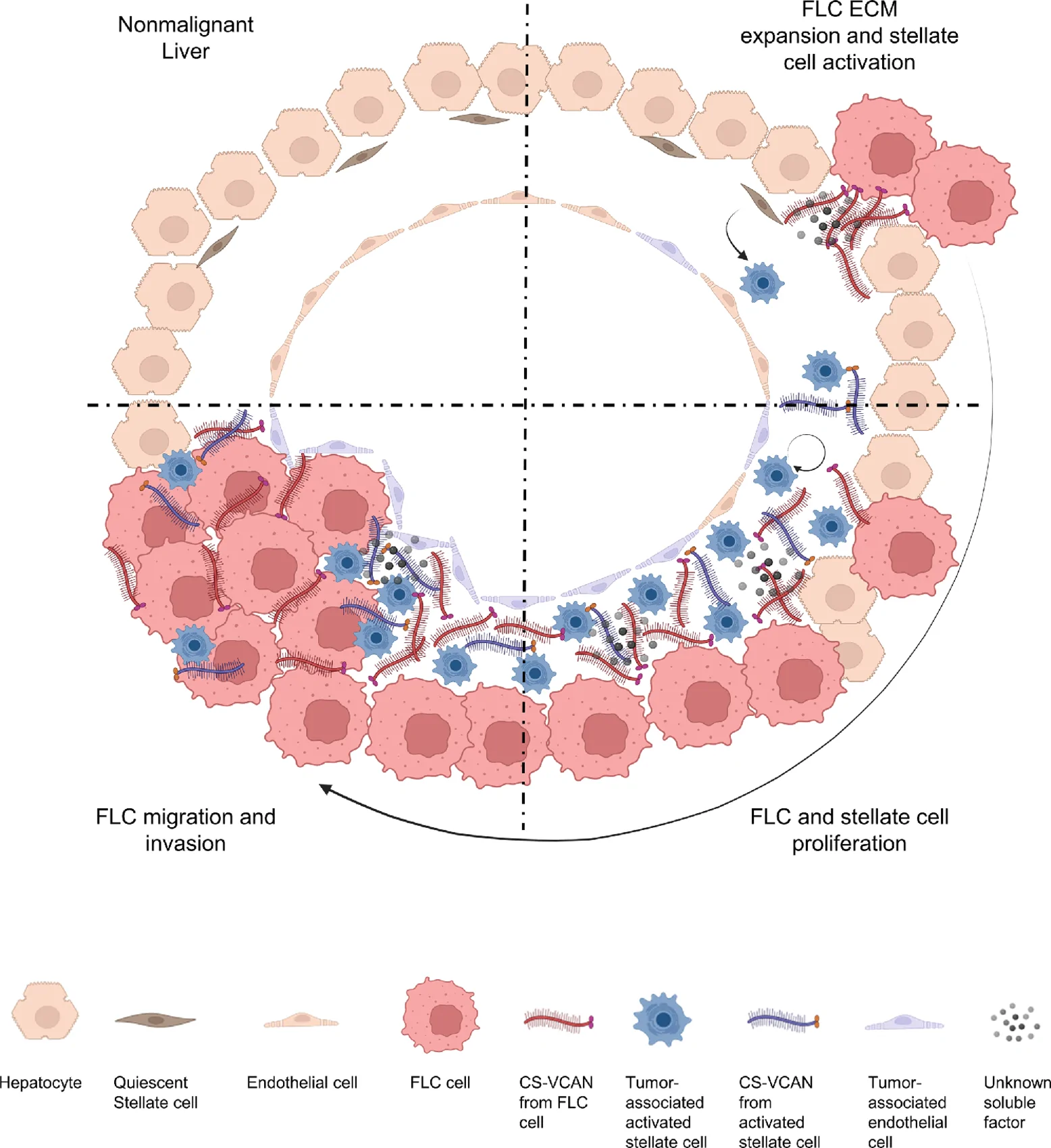
Model for the relevance of CS and VCAN in FLC. A schematic showing the behaviors of nonmalignant hepatocytes, quiescent and activated stellate cells, and endothelial cells with respect to CS and VCAN during FLC progression.

To identify which cells are likely responsible for VCAN production and secretion, we performed scATAC-seq on NML, primary FLC tumor, and metastatic FLC tumor samples (*n* = 3). We used a previously described nuclei isolation protocol (34) and obtained data on nearly 9,500 nuclei total. After data analysis with ArchR (ref. 41; Materials and Methods), nonlinear dimensionality reduction via Uniform Manifold Approximation and Projection (UMAP) revealed eight different clusters (Fig. 6A). By analyzing open chromatin signal at established markers of human liver cell types (53), we assigned each cluster to a specific cell type (Fig. 6A; Supplementary Fig. S4A–D). We also analyzed open chromatin in the deleted region of chromosome 19 to identify the cells that likely harbor the deletion and therefore the DP fusion (Supplementary Fig. S5A and S5B). We then queried for ATAC signal associated with *CSGALNACT1* and detected robust enrichment in the FLC primary and metastatic tumor transformed epithelial cell clusters (Fig. 6B). As expected, there is little to no signal for open chromatin at *CSGALNACT1* in any nonmalignant cell types (Fig. 6B and C). A similar analysis for *VCAN* revealed the strongest signal in tumor-associated activated stellate cells and secondmost in tumor transformed epithelial cells (Fig. 6D and E). These findings suggest that while CS synthesis (via CSGALNACT1) is likely exclusively taking place in tumor transformed epithelial cells, the primary CSAP in FLC, VCAN, is produced and secreted from both activated stellate cells as well as tumor transformed epithelial cells (Fig. 7).

## Discussion

FLC is an aggressive liver cancer that lacks an effective chemotherapeutic remedy. There are several factors contributing to low survival rates in FLC, including vague manifestations, lack of comorbidities, and resistance to general therapeutics. FLC is genetically characterized by the *DNAJB1-PRKACA* (DP) fusion, but efforts to identify specific inhibitors of DP, without targeting wild-type *PRKACA*, have been unsuccessful. In addition, while it is known that DP is sufficient for tumor initiation, it is unclear whether DP expression is essential for tumor maintenance, progression, and metastasis. It has been established in the study of other cancer types that the pericellular environment, including PGs, plays an important role in defining tumor behavior (12, 15). However, to date, no study has investigated GAGs and PGs in FLC. In this study, we sought to bridge this knowledge gap.

The three major classes of GAGs are HA, HS, and CS (3). Of these, only HA is found as a free polymer generated by HA synthetase 1–3 (HAS1–3). The expression levels of these enzymes were found to be extremely low in FLC and, therefore, they were not considered further. HS and CS chains are conjugated to proteins by a shared tetrasaccharide linker. The enzymes responsible for the synthesis of this linker exhibit robust expression in FLC but are unchanged relative to NML tissue. The decision by cells to generate HS or CS sidechains is dependent on stoichiometric competition between the enzymes *N*-acetylglucosaminyltransferase (*EXTL2*), responsible for HS chains, and chondroitin GalNAc transferase (*CSGALNACT1*), responsible for CS chains. We found that the ratio of *CSGALNACT1* to *EXTL2* levels is dramatically elevated in FLC, pointing to CS chains as the key component of the extracellular matrix in FLC.

A major innovation and strength of this study is the development and implementation of a highly sensitive method for quantifying HS and CS disaccharides in patient tissues. This novel assay confirmed that the alterations in expression of CS biosynthetic genes observed in FLC lead to dramatic changes in CS abundance. The quantity of CS in FLC tissue relative to NML was greater than 5.9-fold and the relative difference between CS and HS in tumor tissue was 4.3-fold. In addition, this assay independently quantifies sulfated forms of CS and HS disaccharides. As HS abundance is unchanged in FLC tissue, it is not surprising that there are no significant differences in the seven sulfated forms of HS that we measured. The analysis of sulfated forms of CS showed that nonsulfated CS and two forms of monosulfated CS (CS di-4S and CS di-6S) are significantly increased in FLC tissue. The increased abundance of CS di-4S and CS di-6S is concordant with gene expression increases in associated chondroitin sulfotransferases (*CHST11/3*), again showing that changes in gene expression accurately correspond with changes in chemical abundance. It has been observed in HCC that increased expression of *CHST11/13,* which are functionally equivalent, are upregulated in metastatic samples (54), which may promote sustained Wnt signaling (55). One limitation of the quantitative analysis for the CS chains with di-2S4S should be noted. We were unable to synthesize ^13^C-labeled di2S4S; therefore, the quantitation was completed using the relative quantitation method. However, this should not affect our conclusions, as the levels of di-2S4S are the same in FLC compared with NML tissues.

Given the abundant increase of CS in FLC tissue, and the high expression of the CSAP VCAN, we conjectured that the levels of *CSGALNACT1* and *VCAN* would be correlated. Indeed, we found that there is a positive correlation between the two genes, and between either gene and DP. Consistent with this finding, in a previous report we had demonstrated that the expression of DP in an FLC cell model increases *VCAN* expression (5). We found in this study that the levels of *VCAN* are upregulated in FLC more than in any other cancer type for which expression data are publicly available through TCGA. CCA is the closest to FLC in terms of *CSGALNACT1* and *VCAN* upregulation. Intriguingly, *CHPF* has been reported recently to promote CCA cell growth and invasive potential (56). In addition, *CHSY1* has been reported to suppress apoptosis in colorectal cancer (57) and promote migration in HCC (58).

Another major component of this study is the first-ever snATAC-seq analysis of FLC. All prior genome-scale analyses of FLC have been performed on bulk tumors and the only published single-nucleus analysis related to this cancer involves a patient-derived xenograft, not a primary tumor (59). Our study overcomes these limitations and provides the first glimpse into FLC tumor tissue complexity. We focused on resolving gene locus activity at single-nucleus resolution and identified chromatin accessibility at the *CSGALNACT1* and *VCAN* loci in the primary population of FLC cells. Transformed epithelial cells were identified as the primary source of *CSGLANACT1* locus activity, whereas proliferating stellate cells were found to be the cell type with the strongest *VCAN* signal. This finding suggests that communication between transformed DP+ liver epithelial cells and stellate cells may be critical to FLC disease progression.

The expression and secretion of VCAN from activated stellate cells is a normal response to liver injury (60–63). The data generated in this study suggest that FLC cells upregulate *CSGALNACT1* and *VCAN* in a DP-dependent manner and begin secreting CS-VCAN PG into the extracellular matrix. The increased accumulation of VCAN may sequester higher concentrations of growth factors, or increase mechanical tension, and induce the activation of quiescent stellate cells. Upon activation, these stellate cells proliferate and secrete VCAN as a normal response to a perceived injury. VCAN is a highly modular protein containing four distinct domains (G1, GAGα, GAGβ, and G3). The G1 and G3 domains govern direct interactions with extracellular matrix components and cell surfaces, such as with HA and EGF receptor, respectively. The GAGα and GAGβ domains contain sites for CS chain conjugation. Through alternative splicing, six distinct isoforms, V0–4 and versikine (a secreted version), have been described. Only the V0 isoform contains all four domains and V1 and V2 contain GAGβ or GAGα, respectively. The V3, V4, and versikine isoforms are smaller peptides and contain little-to-no sites for CS conjugation. The CS containing isoforms V0, V1, and V2 may also differ across cell types in terms of degree of CS conjugation, elongation, and sulfation, all which affect interactions with soluble factors. Because of these variables, VCAN can promote pleiotropic downstream effects; therefore, determining the specific functions of VCAN in cancer is not trivial (64, 65). Identifying which protein isoforms of VCAN are expressed in FLC tumor epithelial cells and activated stellate cells is a critical next step toward defining the role of VCAN in FLC progression.

The role for stellate cells to promote fibrosis and predispose the liver to cancer formation is well established (66) and multiple HS PGs have been implicated in this role, including syndecans (67–70), glypicans (71–73), and even free HS disaccharides (74). Activated stellate cells can promote the formation of CCA (75), a characteristically desmoplastic tumor. Our finding that *CSGALNACT1* and *VCAN* expression levels in FLC are most similar to that of CCA suggests potential mechanistic parallels between the two cancer types in terms of fibrosis. In addition, CS PGs, including VCAN and CD44, have been implicated in both hepatic fibrosis and HCC formation (31, 76–78). However, these findings in HCC suggest that stellate-mediated fibrosis precedes and contributes to cancer formation. Given that patients with FLC lack preceding fibrotic conditions, such as cirrhosis, the relationship between stellate activation, fibrosis, and cancer development in FLC and CCA may be fundamentally different relative to HCC. Further investigation is required to define the roles of VCAN in tumor proliferation and invasion, either by direct influence on tumor epithelial cells, or by indirect means such as communication with activated stellate cells to affect the ECM, or both. These follow-up studies may also reveal whether VCAN is compelling as a direct therapeutic target in FLC.

We have implemented several novel methods to provide the first high-resolution analysis of PG biology in FLC tumors. Our findings motivate further investigation of VCAN in FLC progression. Future research may also study the effects of VCAN inhibitors on FLC cell drug resistance, growth and/or metastasis.

## Supplementary Material

Supplementary Data Figures S1-S5 All supplemental figures with legends.
