# Supplementary material for: Chemical, Molecular, and Single-nucleus Analysis Reveal Chondroitin Sulfate Proteoglycan Aberrancy in Fibrolamellar Carcinoma
Source: Cancer Res Commun. 2022 Jul 18;2(7):663–78. doi: 10.1158/2767-9764.CRC-21-0177 (PMC10010304; doi:10.1158/2767-9764.CRC-21-0177)
Supplement: Supplementary Data Figures S1-S5 — All supplemental figures with legends. [file crc-21-0177-s01.docx]

**
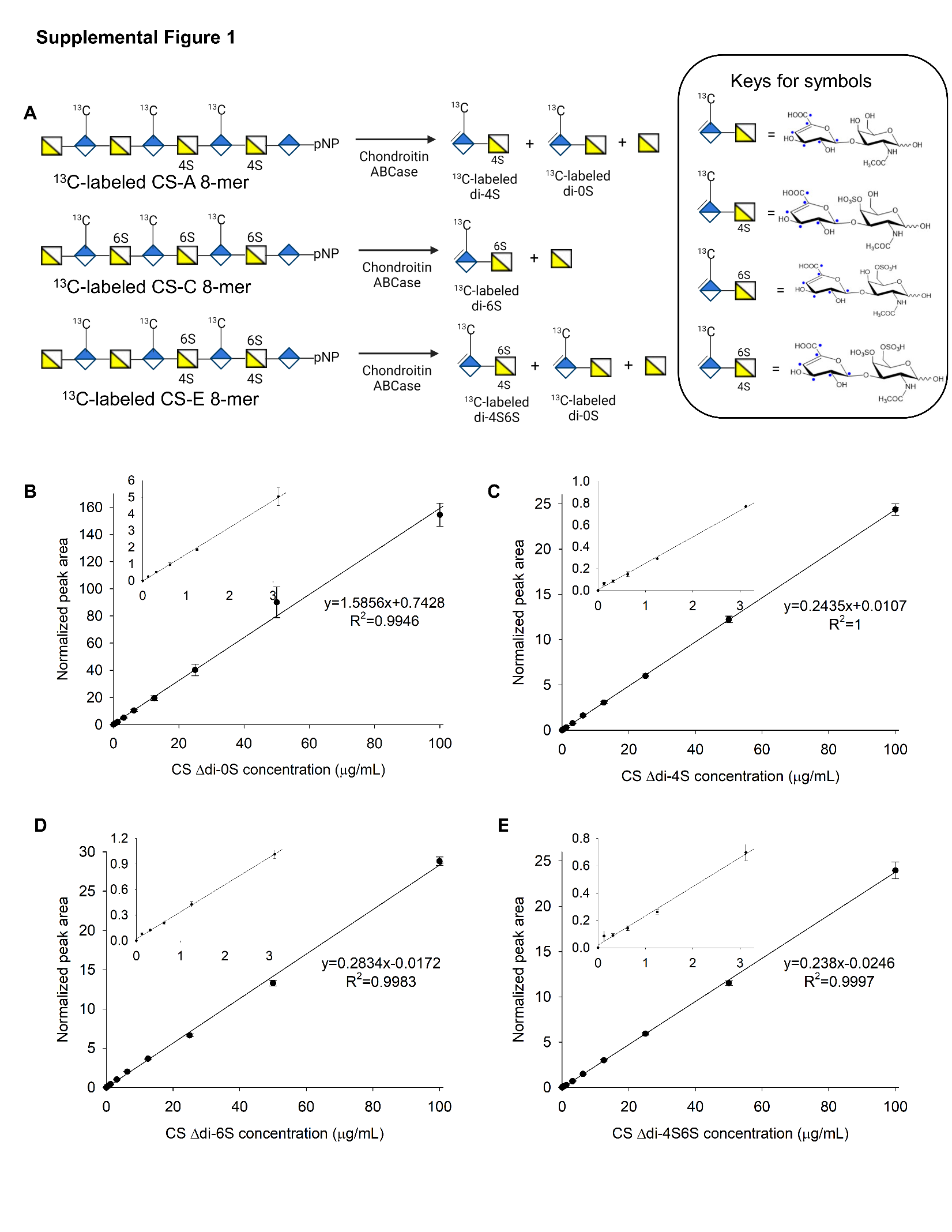
Supplemental Figure 1. Quantification of chondroitin sulfate disaccharides. (A)** Schematic of preparation of ^13^C-labeled CS disaccharides. Four ^13^C-labeled CS disaccharide calibrants were prepared from chondroitin ABCase digestion of three ^13^C-labeled 8-mers. The disaccharides were purified to homogeneity after a Q-Sepharose column purification. The ^13^C-labeled carbon atoms in the DUA residue are indicated with blue dots. pNP represents p-nitrophenyl. **(B)** The curve and linear equation of normalized peak area as a function of concentration for ∆di-0S are shown. The concentration of ∆di-0S used for LC-MS/MS analysis were 0.125, 0.313, 0.625, 1.25, 3.13, 6.25, 12.5, 25, 50 and 100 μg/mL, mixing with 0.8 μg/mL disaccharide calibrant ∆di-4S. Data represent means ± S.D. (n=3) **(C)** The curve and linear equation of normalized peak area as a function of concentration for ∆di-4S are shown. The concentration of ∆di-4S used for LC-MS/MS analysis were 0.125, 0.313, 0.625, 1.25, 3.13, 6.25, 12.5, 25, 50 and 100 μg/mL, mixing with 4 μg/mL disaccharide calibrant ∆di-4S. Data represent means ± S.D. (n=3) **(D)** The curve and linear equation of normalized peak area as a function of concentration for ∆di-6S are shown. The concentration of ∆di-6S used for LC-MS/MS analysis were 0.125, 0.313, 0.625, 1.25, 3.13, 6.25, 12.5, 25, 50 and 100 μg/mL, mixing with 4 μg/mL disaccharide calibrant ∆di-6S. Data represent means ± S.D. (n=3) **(E)** The curve and linear equation of normalized peak area as a function of concentration for ∆di-4S6S are shown. The concentration of ∆di-4S6S used for LC-MS/MS analysis were 0.125, 0.313, 0.625, 1.25, 3.13, 6.25, 12.5, 25, 50 and 100 μg/mL, mixing with 4 μg/mL disaccharide calibrant ∆di-4S6S. Data represent means ± S.D. (n=3)

**
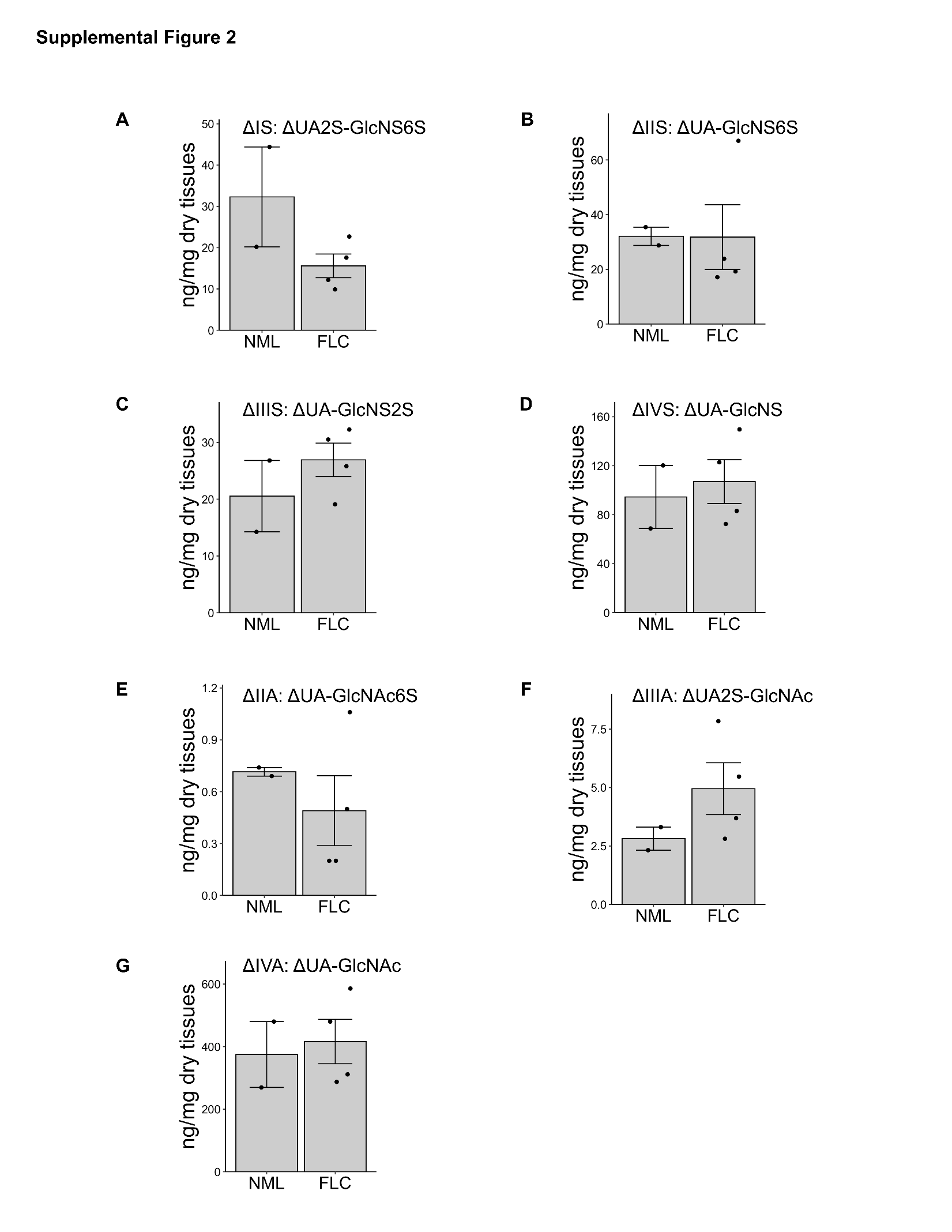
Supplemental Figure 2. Quantification of heparan sulfate disaccharides. (A)** Nanograms of HS ΔUA2S-GlcNS6S (ΔIS) per milligram of dry tissue in FLC (n=4) and NML (n=2) tissue. **(B)** Nanograms of HS ΔUA-GlcNS6S (ΔIIS) per milligram of dry tissue in FLC (n=4) and NML (n=2) tissue. **(C)** Nanograms of HS ΔUA-GlcNS (ΔIIIS) per milligram of dry tissue in FLC (n=4) and NML (n=2) tissue. **(D)** Nanograms of HS ΔUA-GlcNS (ΔIVS) per milligram of dry tissue in FLC (n=4) and NML (n=2) tissue. **(E)** Nanograms of HS ΔUA-GlcNAc6S (ΔIIA) per milligram of dry tissue in FLC (n=4) and NML (n=2) tissue. **(F)** Nanograms of HS ΔUA2S-GlcNAc (ΔIIIA) per milligram of dry tissue in FLC (n=4) and NML (n=2) tissue. **(G)** Nanograms of HS ΔUA-GlcNAc (ΔIVA) per milligram of dry tissue in FLC (n=4) and NML (n=2) tissue.

**
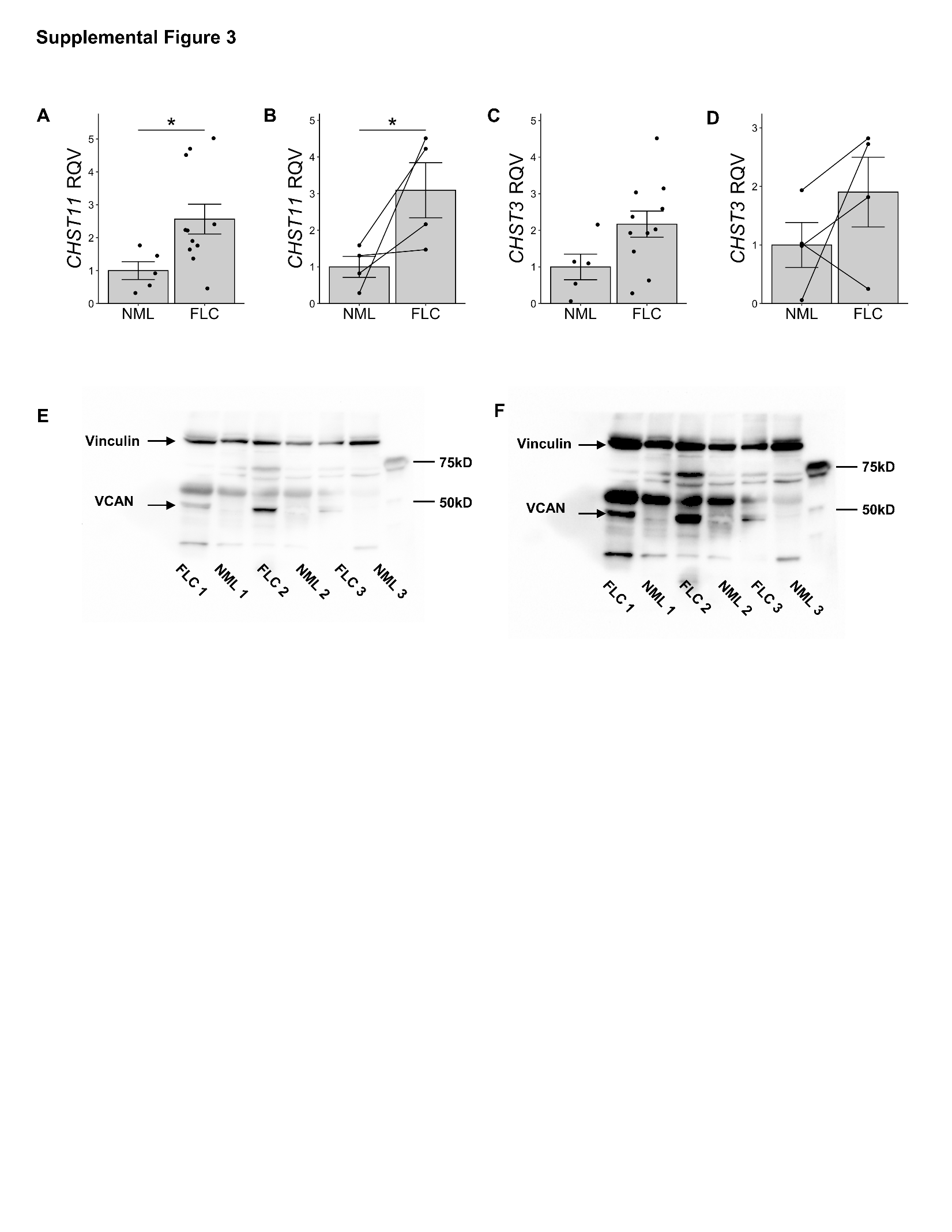
Supplemental Figure 3. Additional gene expression analysis in FLC samples and additional VCAN immunoblots. (A)** Quantitative PCR showing the relative quantitative value (RQV) of *CHST11* in a subset of FLC samples that have matched NML tissue (n=4). **(B)** The matched NML/FLC samples are indicated with a line linking the two data points. **(C)** Quantitative PCR showing the relative quantitative value (RQV) of *CHST3* in a subset of FLC samples included in this study (n=11) compared to NML samples (n=5). **(D)** Quantitative PCR showing the relative quantitative value (RQV) of *CHST3* in a subset of FLC samples that have matched NML tissue (n=4). The matched NML/FLC samples are indicated with a line linking the two data points. **(E)** Short and **(F)** long exposure VCAN immunoblot from Figure 4D shown in entirety. Vinculin and versican protein are denoted with arrows and molecular weight standards are denoted by dashes. Samples are labeled at the bottom of the panel. The short exposure blot was used for quantification in Figure 4E. **P*<0.05, two-tailed Student’s t-test.

**
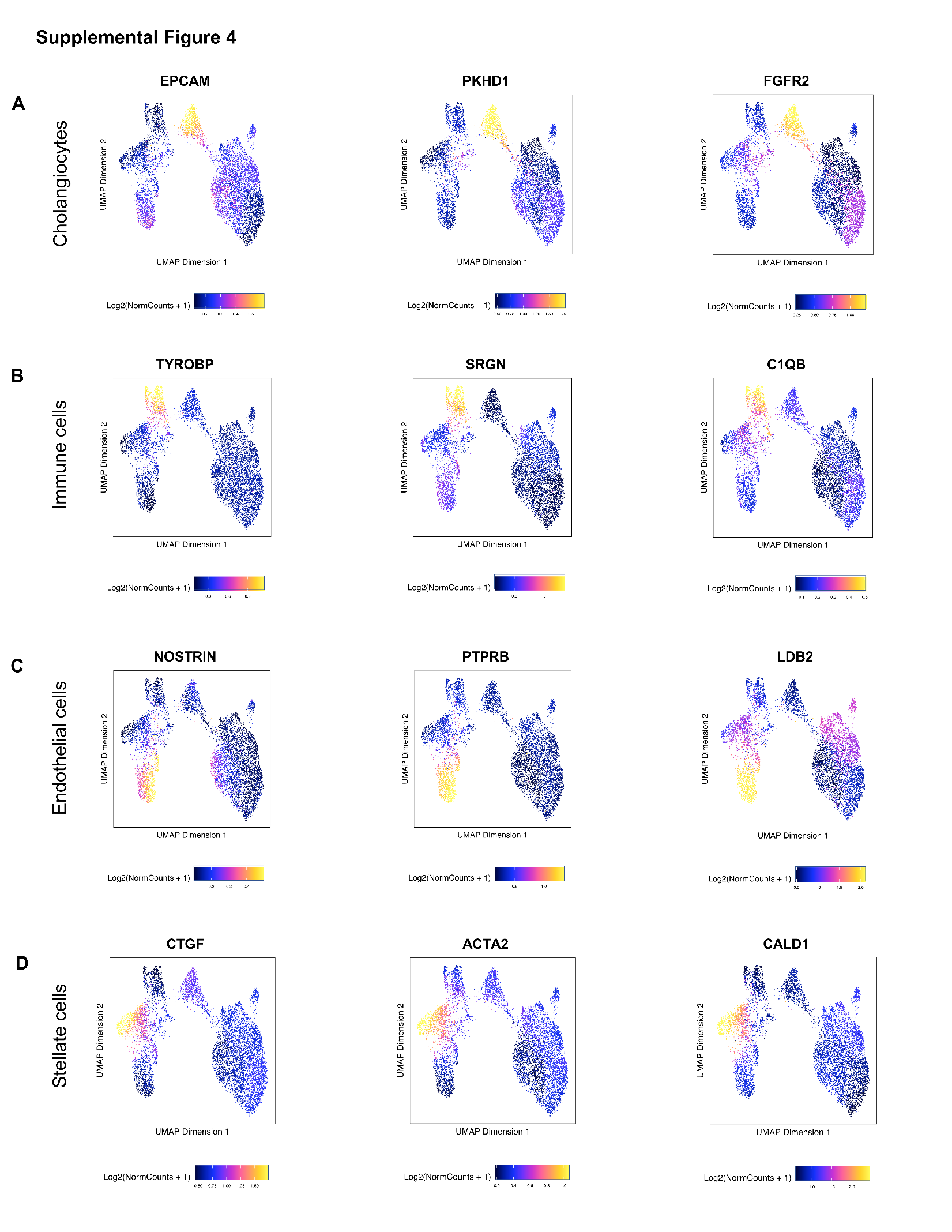
Supplemental Figure 4. Marker genes of cell clusters from the single-cell ATAC data. (A)** Signal intensity of chromatin accessibility near the *EPCAM*, *PKHD1*, and FGFR2 gene loci denoting cholangiocytes. **(B)** Signal intensity of chromatin accessibility near the *TYROBP*, *SRGN*, and *C1QB* gene loci denoting immune cells. **(C)** Signal intensity of chromatin accessibility near the *NOSTRIN*, *PTPRB*, and *LDB2* gene loci denoting endothelial cells. **(D)** Signal intensity of chromatin accessibility near the *CTGF*, *ACTA2*, and *CALD1* gene loci denoting stellate cells. Increasing signal is indicated by the color gradient (maximum signal is yellow and minimal signal is dark blue).

**
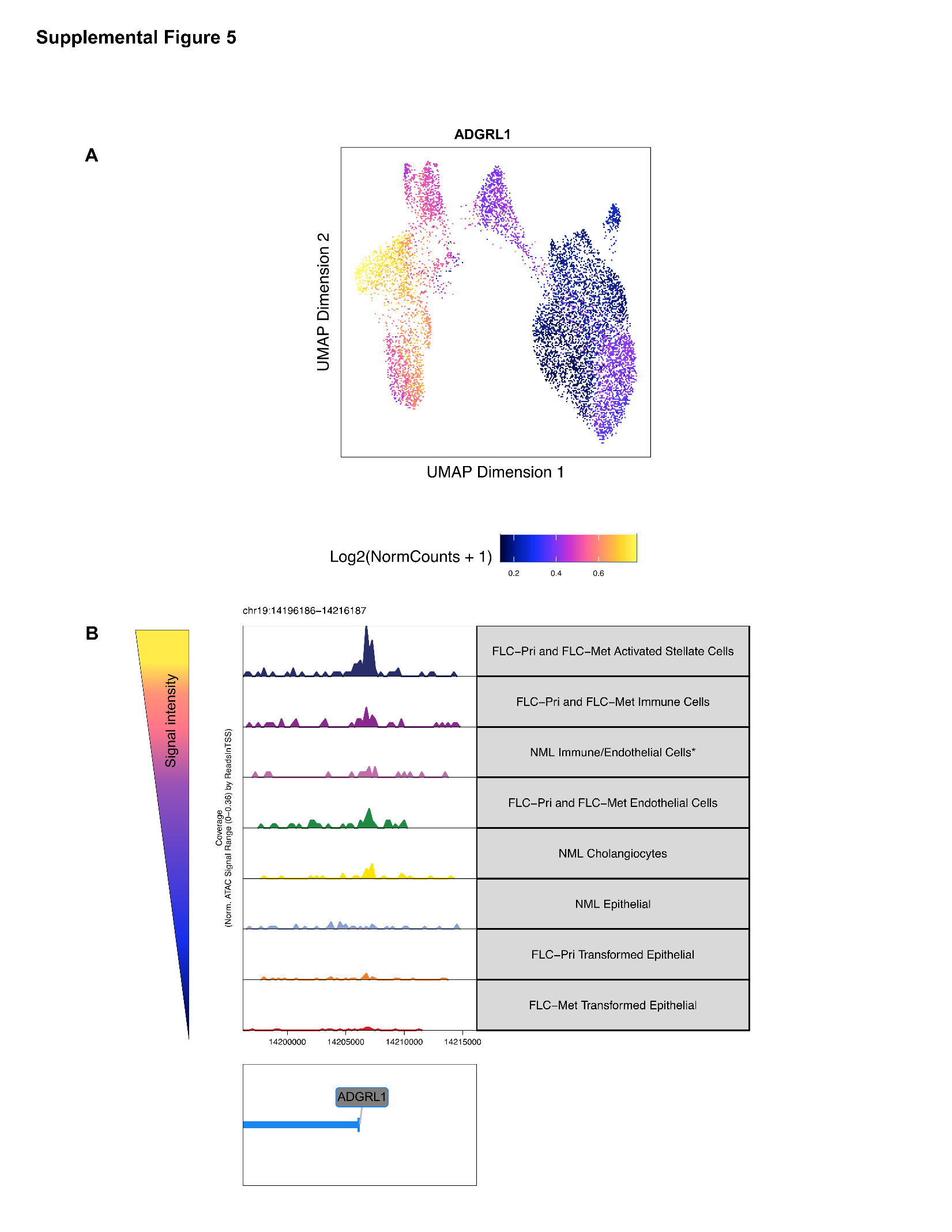
Supplemental Figure 5. Identification of cells that are likely positive for the signature deletion event in FLC. (A)** Signal intensity of chromatin accessibility near the *ADGRL1* gene locus. **(B)** Genome tracks showing the location of open chromatin signal near the *ADGRL1* locus in each cell type. The annotated transcriptional start site for *ADGRL1* is shown at the bottom of the panel.
